# Synergistic Mn‐MOF Activation of Pistol Ribozymes for Cancer Immunotherapy

**DOI:** 10.1002/advs.202517912

**Published:** 2026-02-10

**Authors:** Ming Zhao, Shan Qiao, Jie Bai, Jingjing Zhang, Zhiqin Xi, Zhenzhen Li, Xuelin Zhan, Ying Zhang, Xiaotong Yu, Yao Chen, Yijin Liu

**Affiliations:** ^1^ State Key Laboratory of Medicinal Chemical Biology and College of Pharmacy Nankai University Tianjin China; ^2^ Frontiers Science Center for Cell Responses Frontiers Science Center for New Organic Matter and Tianjin Key Laboratory of Molecular Drug Research Nankai University Tianjin P. R. China; ^3^ Guangzhou National Laboratory Guangzhou International Bio Island Guangzhou Guangdong P. R. China; ^4^ China Regional Research Centre International Centre for Genetic Engineering and Biotechnology Taizhou Jiangsu P. R. China; ^5^ School of Medicine University of St Andrews St Andrews Fife UK; ^6^ State Key Laboratory of Biopharmaceutical Preparation and Delivery Institute of Process Engineering Chinese Academy of Sciences Beijing P. R. China

**Keywords:** gene‐targeted therapy, manganese ions, metal−organic frameworks (MOFs), nucleic acid drug delivery, pistol ribozyme

## Abstract

Checkpoint blockade therapies targeting PD‐L1 have revolutionized cancer immunotherapy, yet their efficacy is constrained by systemic immune toxicity and inadequate immune infiltration in certain tumor types. Here, we introduce a synergistic gene‐silencing nanosystem based on a target‐selective Pistol ribozyme (PS473) encapsulated within a manganese‐based, pH‐responsive metal–organic framework (NKMOF‐101‐[Mn]). The engineered PS473 exhibited high cleavage efficiency toward GU‐rich PD‐L1 mRNA motifs and was further activated by Mn^2^^+^ cofactors. NKMOF‐101‐[Mn] not only protects the ribozyme from nuclease degradation but also enables localized Mn^2^^+^ release to increase catalytic activity and innate immune signaling under the acidic tumor microenvironment. In vitro, PS473@NKMOF‐101‐[Mn] markedly suppressed PD‐L1 expression and promoted macrophage activation. In the B16F10 melanoma model, this system achieved over 90% tumor inhibition, enhanced immune cell infiltration and activation, and exhibited minimal systemic toxicity. Transcriptomic profiling further revealed the upregulation of immune‐related pathways, supporting a synergistic mechanism of gene silencing and immune activation. Overall, this study established a ribozyme‐directed immunotherapeutic platform with strong potential for precision cancer therapy via checkpoint modulation and immune reprogramming.

## Graphical abstract

A Mn^2^^+^‐based metal–organic framework delivers a catalytic Pistol ribozyme for targeted PD‐L1 mRNA cleavage in tumor cells, with the released Mn^2^^+^ not only inducing immune activation in immune cells but also increasing the Pistol ribozyme catalytic activity. This dual mechanism—checkpoint silencing (via activated ribozyme‐mediated PD‐L1 mRNA cleavage) and innate immune activation—synergistically enhances antitumor immunity and promotes efficient immune‐mediated tumor cell elimination.

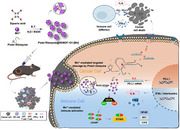

## 1 Introduction

Cancer immunotherapy has revolutionized clinical treatment by harnessing the body's immune system to combat tumors [1, 2, 3]. However, its efficacy remains limited in many patients because of the immunosuppressive tumor microenvironment (TME) and immune checkpoint pathways, such as the PD‐1/PD‐L1 axis [4, 5, 6, 7]. Targeted gene silencing of immunosuppressive checkpoints within tumor cells represents a promising route to reinvigorate antitumor immunity [8, 9, 10, 11, 12].

Despite major advances in RNA therapeutics—including siRNAs [13], antisense oligonucleotides [14], and mRNA vaccines [15]—catalytic ribozymes remain underexplored as therapeutic molecules, largely due to their susceptibility to nucleases, dependence on divalent metal ions, and limited strategies for efficient intracellular delivery. Recent progress in RNA structural biology and chemical engineering has renewed interest in small self‐cleaving ribozymes as programmable tools for precise post‐transcriptional gene regulation [16, 17, 18, 19].

Among catalytic RNAs, Pistol ribozymes are particularly appealing due to their compact architecture, robust folding, and metal‐ion‐responsive catalytic properties [16]. These self‐cleaving RNA enzymes employ a general acid–base mechanism involving water and divalent metal ions such as Mg^2^^+^, enabling robust and programmable cleavage activity (Figure S1A) [20, 21]. Notably, recent biochemical studies have shown that Pistol ribozymes exhibit higher catalytic activity in Mn^2^^+^ compared with Mg^2^^+^ [22], suggesting that the catalytic landscape of these ribozymes can be modulated by metal ions. However, their therapeutic application remains largely unexplored, especially in the context of tumor immune evasion [23] and in vivo RNA delivery [24, 25].

**FIGURE 1:**
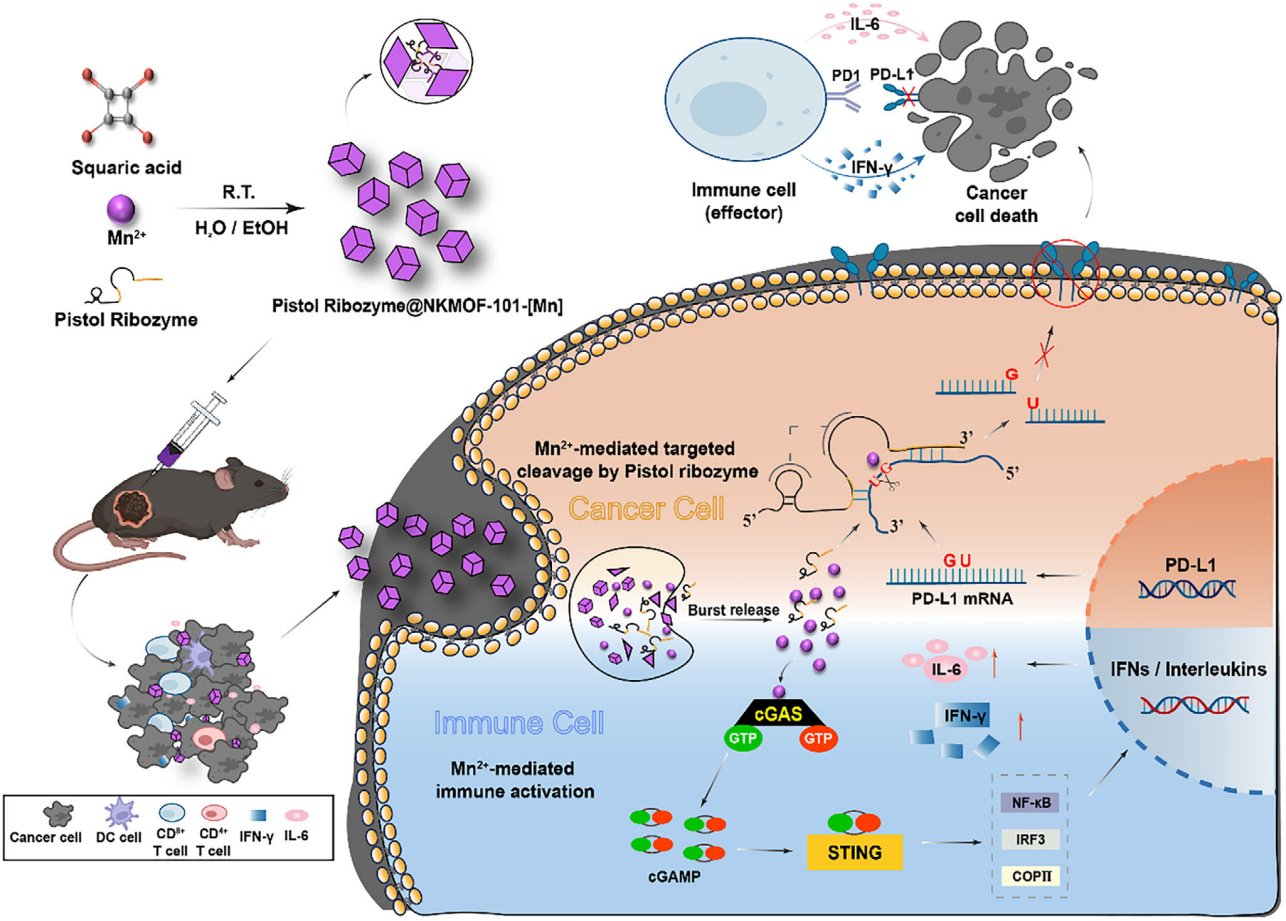
Schematic illustration of the PS473@NKMOF‐101‐[Mn] nanosystem enabling synergistic cancer immunotherapy. The system integrates a catalytic Pistol ribozyme into Mn^2^^+^‐based metal‒organic framework nanoparticles (NKMOF‐101‐[Mn]) to achieve targeted cleavage of PD‐L1 mRNA in cancer cells. Upon internalization by tumor cells, the nanosystem rapidly releases Mn^2^^+^ and the ribozyme, leading to PD‐L1 mRNA silencing and reduced PD‐L1 expression. Concurrently, Mn^2^^+^ activates the innate immune cGAS‒STING pathway in immune cells, promoting the secretion of interferons and interleukins. The combined effects of PD‐L1 silencing and immune activation enhance cytotoxic T‐cell‐mediated tumor cell killing.

To enable localized checkpoint silencing while simultaneously engaging innate immune pathways, we designed a nanosystem (PS473@NKMOF‐101‐[Mn]) in which a PD‐L1–targeting Pistol ribozyme is encapsulated within a Mn^2^^+^‐based nanoscale metal–organic framework (NKMOF‐101‐[Mn]) (Figure 1). The nanocarrier protects the ribozyme and facilitates its intracellular delivery, while also serving as a depot for controlled Mn^2^^+^ release. Upon cellular uptake, the system provides both the ribozyme and bioavailable Mn^2^^+^—a metal ion known to influence the catalytic behavior of Pistol ribozymes and to participate in innate immune signaling pathways such as cGAS–STING activation [26, 27, 28]. Importantly, Mn^2^^+^‐based nanomaterials have been increasingly explored in cancer immunotherapy, where controlled and localized release has been shown to achieve immune activation without systemic toxicity [29, 30, 31, 32, 33, 34]. Building on these advances, our platform is designed to integrate RNA‐based checkpoint silencing with metal‐dependent immunomodulation in a single nanoscale formulation.

## 2 Results

### 2.1 Design and Assembly of Pistol Ribozymes Targeting PD‐L1

**FIGURE 2:**
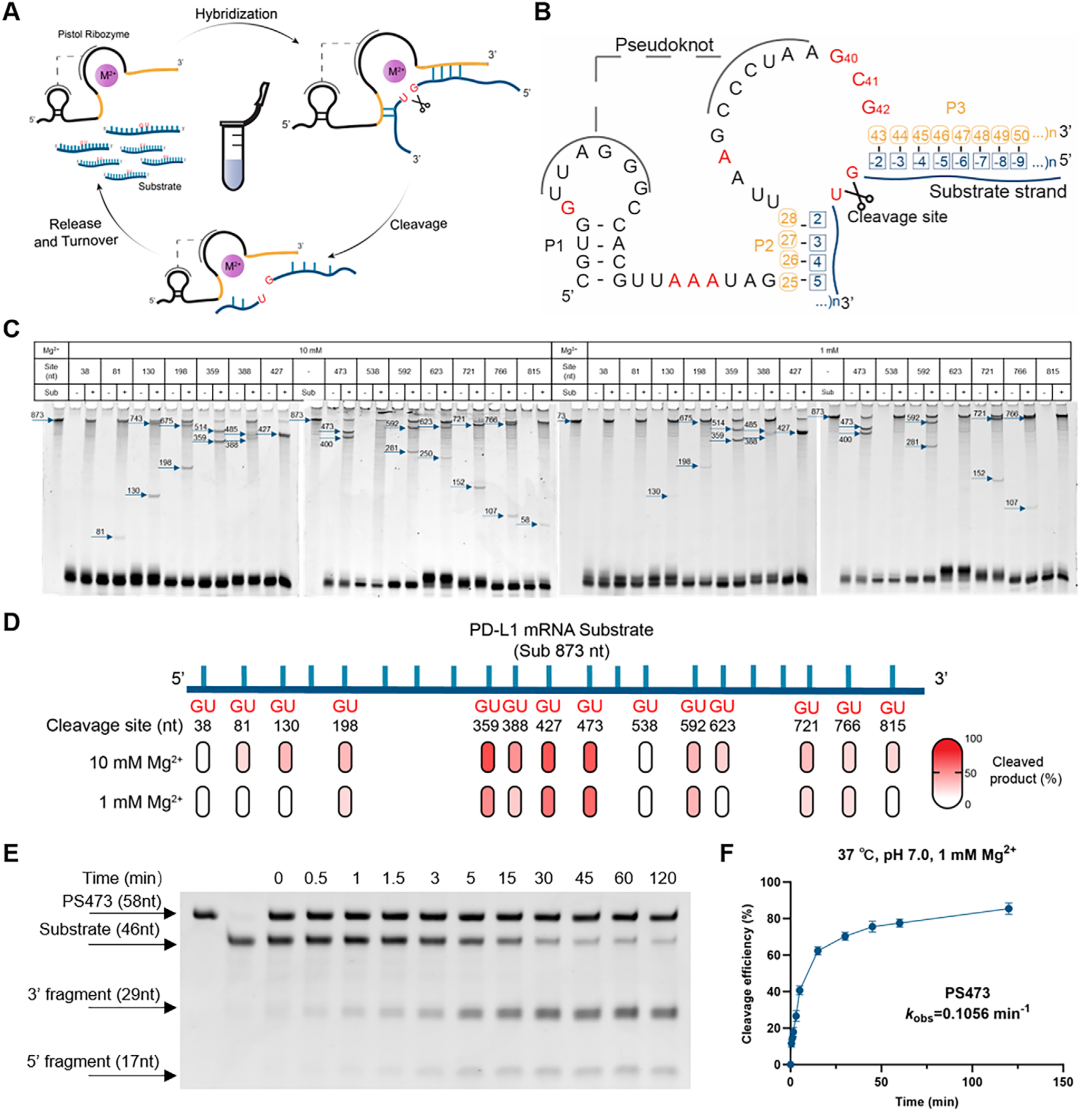
Structural design and catalytic characterization of the PD‐L1–targeting Pistol ribozyme. (A) Schematic diagram illustrating the catalytic cycle of Pistol ribozyme, which involves substrate hybridization, site‐specific cleavage, and turnover via product release. The ribozyme cleaves the target RNA strand at a defined GU site in the presence of divalent metal ions. (B) Predicted secondary structure of Pistol ribozyme, highlighting conserved nucleotides (in red) and the pseudoknot motif. The cleavage site is indicated. (C) Representative gel electrophoresis analysis showing the cleavage of the PD‐L1 mRNA fragment by Pistol ribozyme variants under 10 and 1 mM Mg^2^^+^. (D) Mapping of potential GU dinucleotide sites and assessment of Pistol ribozyme cleavage efficiency across the PD‐L1 mRNA coding region corresponding to the substrate used in (C). Red shading indicates the percentage of cleavage products. (E) Time‐course denaturing PAGE analysis of a representative PS473‐catalyzed cleavage reaction of a 46‐nt PD‐L1 RNA fragment under near‐physiological conditions (37°C, pH 7.0, 1 mM Mg^2^^+^). The intact substrate (46 nt) and cleavage products (29 and 17 nt) are resolved over a 120‐min reaction. (F) Kinetic profile of PS473‐mediated cleavage quantified from (E) and fitted to a single‐exponential model, yielding an observed rate constant (*k*_obs_ = 0.1056 min^−1^). The error bars represent the standard deviation (± SD); *n = 3*.

Guided by secondary structure predictions and the conserved catalytic motifs of active Pistol ribozymes, we designed a library of ribozyme variants adopting a stem–loop architecture with 3D pseudoknot interactions. These structural features promote compact global folding and efficient recognition of GU dinucleotide motifs within RNA substrates (Figure 2A,B). The ribozymes were engineered to cleave GU sites distributed along the coding sequence (CDS) of PD‐L1 mRNA (NM_021893.3), thereby enabling site‐specific posttranscriptional gene silencing (Table S1). A catalytically inactive mutant (M5; Figure S1B–G and Table S2) was also generated and used as a negative control in subsequent experiments. Detailed design information can be found in the Methods and Supplementary Methods sections.

**FIGURE 3:**
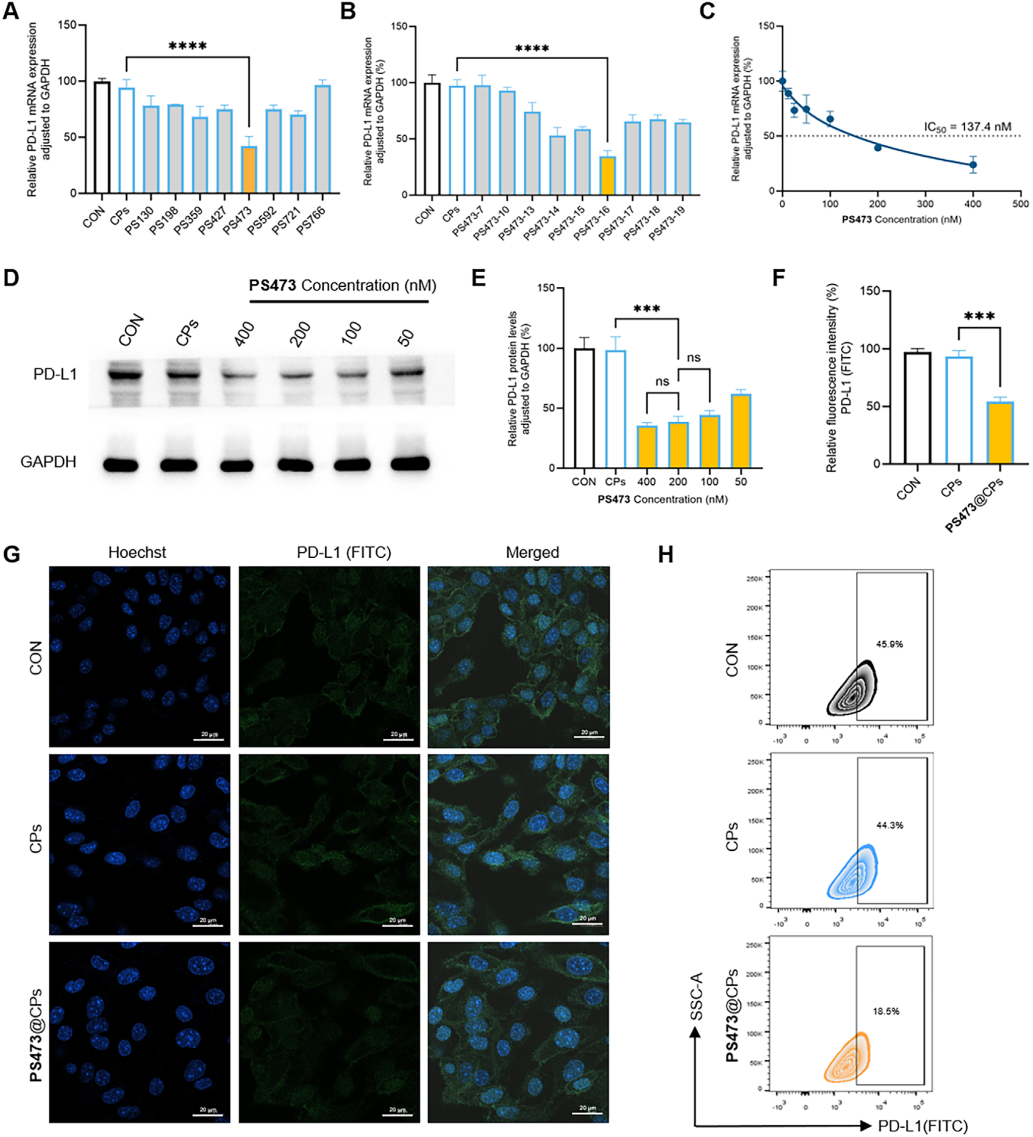
Cellular evaluation of PS473‐mediated PD‐L1 gene silencing. (A,B) Relative suppression efficiency of Pistol ribozyme variants on PD‐L1 mRNA expression in B16F10 cells. CPs = cationic polymers. (C) qRT‒PCR analysis showing a dose‐dependent reduction in PD‐L1 mRNA levels in B16F10 cells treated with increasing concentrations of the optimized ribozyme PS473‐16 (PS473), calculated half‐maximal inhibitory concentration (IC_50_ = 137.4 nM). (D,E) Western blot analysis (D) and densitometric quantification (E) showing a dose‐dependent decrease in PD‐L1 protein levels following PS473‐16 (PS473) treatment. (F,G) Quantitative analysis (F) and immunofluorescence imaging (G) showing PD‐L1 expression (green) and nuclei (blue). (H) Representative flow cytometry contour plots illustrating the expression of PD‐L1 surface protein in the indicated groups, with distinct cell population distributions reflecting differences in PD‐L1 expression levels. Data represent mean ± SD from three independent experiments (*n = 3*). Exact *p* values reported as follows: ^***^ *p* < 0.001, ^****^ *p* < 0.0001.

Because Mg^2^^+^ represents the predominant intracellular cofactor for small self‐cleaving ribozymes, the initial in vitro cleavage screening was performed under physiologically relevant Mg^2^^+^ concentrations to identify variants with robust baseline activity before evaluating their metal‐ion dependence. In vitro cleavage screening revealed that several constructs exhibited robust catalytic activity at specific target sites (Figure 2C), with cleavage efficiency increasing in a concentration‐dependent manner with Mg^2^^+^ (Figure 2D). Among these, four ribozymes targeting positions 359, 388, 427, and 473 showed particularly high activity under 10 mM Mg^2^^+^ conditions. Although multiple constructs exhibited comparable in vitro performance, the variant targeting the GU site at position 473 (designated PS473) produced the most pronounced PD‐L1 silencing in cells (Figure 3A; Figure S1I), and was therefore selected as the lead candidate for further optimization. PS473 retained strong activity under quasiphysiological conditions (1 mM Mg^2^^+^, pH 7.0, 37°C), achieving over 80% substrate cleavage within 2 h with a kinetic rate constant of *k*_obs_ = 0.1056 min^−^^1^ (Figure 2E,F). To further optimize its activity, we systematically screened PS473 variants with different P3 stem lengths. This analysis identified the PS473‐16 variant (PS473), which forms 16 base pairs with the substrate in the P3 region, as the most efficient construct in vitro (Figure S1H). BLAST analysis against the mouse transcriptome revealed that PS473‐16 possesses no detectable off‐target complementarity outside PD‐L1, indicating a high degree of sequence specificity.

### 2.2 Pistol Ribozymes Effectively Silence PD‐L1 Expression in Cells

We next evaluated the gene‐silencing efficacy of the PS473 series variants (Table S3) of Pistol ribozymes in cell‐based assays. Quantitative PCR analysis revealed that among the tested designs, PS473‐16 (PS473) exhibited the strongest suppression of PD‐L1 mRNA in cells (Figure 3A,B; Figure S1I). A clear dose‒dependent reduction was observed, with increasing concentrations of PS473‐16 (PS473) leading to progressive reductions in PD‐L1 mRNA and protein levels (Figure 3C–E). Specifically, treatment with 200 nM PS473‐16 resulted in approximately 60% knockdown of PD‐L1 mRNA, with higher doses yielding over 80% knockdown. At the protein level, both the 200 and 400 nM treatments yielded similarly robust suppression. To identify the optimal working concentration, we analyzed the dose–response curve and determined a half‐maximal inhibitory concentration (IC_50_) of 137.4 nM (Figure 3C). It is also noteworthy that at a concentration of 200 nM, the catalytically inactive M5 construct, which shares the same substrate recognition domain as PS473‐16 (PS473), exhibited no significant knockdown effect on PD‐L1 mRNA (Figure S1G).

Accordingly, 200 nM—a concentration above the IC_50_ yet within a biologically safe range—was selected as the standard treatment concentration to balance silencing efficiency with biocompatibility and minimize potential cytotoxicity caused by excessive exogenous RNA.

The effect of PS473‐16 (PS473) on PD‐L1 protein expression was further validated by immunofluorescence staining of B16F10 cells with FITC‐conjugated anti‐mouse PD‐L1 antibodies. Confocal microscopy and fluorescence quantification revealed an approximate 50% decrease in PD‐L1 protein levels following PS473‐16 (PS473) treatment relative to untreated controls (Figure 3F,G). Consistently, flow cytometry analysis showed a marked decline in the proportion of PD‐L1–positive cells from 44.3% to 18.5% after PS473‐16 exposure (Figure 3H).

Together, these findings demonstrate that PS473‐16 (hereafter referred to as PS473) is a potent and specific ribozyme capable of efficiently silencing PD‐L1 expression at both the mRNA and protein levels in tumor cells.

### 2.3 Mn^2^^+^ Significantly Enhances the Catalytic Activity of Pistol Ribozymes

**FIGURE 4:**
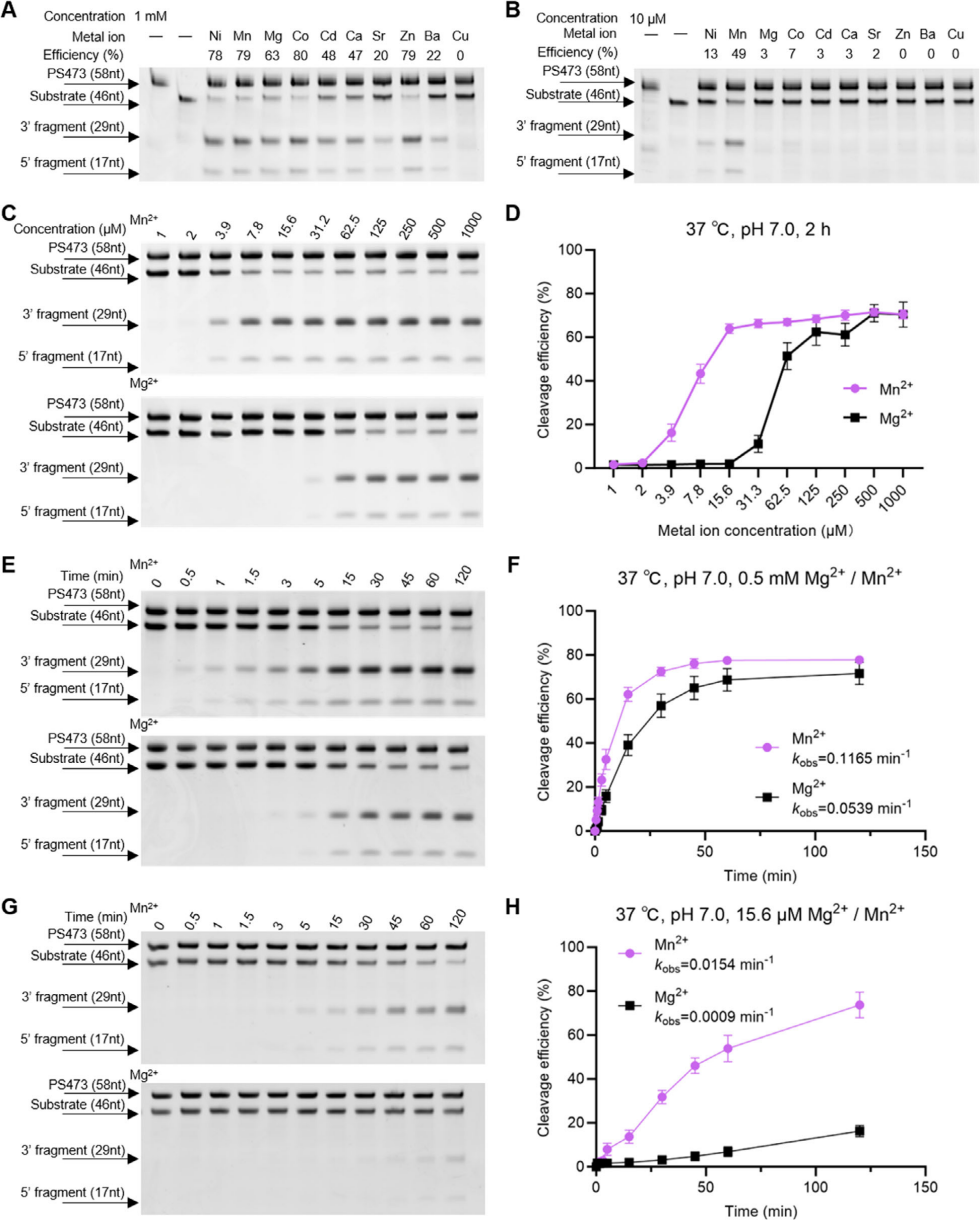
Metal ion–dependent catalytic activity of the PS473 ribozyme. (A,B) Representative gel electrophoresis showing the cleavage of 46nt PD‐L1 RNA substrate by PS473 in the presence of different divalent metal ions at a concentration of 1 mM (A) or 10 µM (B) in 2 h. The cleaved 5′ and 3′ fragments are indicated. (C) Representative gel electrophoresis showing the cleavage of 46nt PD‐L1 RNA substrate by PS473 under Mg^2^^+^ or Mn^2^^+^ concentrations ranging 0–1000 µM. (D) Quantification of cleavage efficiency from (C), demonstrating markedly greater catalytic activity in the presence of Mn^2^^+^ than in the presence of Mg^2^^+^ across a broad concentration range. (E–H) Time‐course cleavage of 46nt PD‐L1 RNA substrate by PS473 in the presence of Mn^2^^+^ or Mg^2^^+^ under different metal ion concentrations. At 0.5 mM (E,F), both cations supported cleavage activity, with Mn^2^^+^ yielding a higher rate constant (*k*_obs_ = 0.1165 min^−^^1^) than Mg^2^^+^ (*k*_obs_ = 0.0539 min^−^^1^). At a lower concentration of 15.6 µM (G,H), Mn^2^^+^ maintained substantially greater catalytic efficiency, whereas Mg^2^^+^ activity was minimal (*k*_obs_ = 0.0154 vs. 0.0009 min^−^^1^). All reactions were conducted at 37°C in pH 7.0 buffer (5 mM HEPES, 20 mM NaCl). Data represent mean ± SD (*n* = 3).

Previous studies have demonstrated that Pistol ribozymes display differential catalytic activities in the presence of various divalent metal ions, with Co^2^^+^ and Mn^2^^+^ supporting higher activity than Mg^2^^+^ [22, 35]. To assess the metal‐ion dependence of PS473 and determine how different divalent cations influence its intrinsic catalytic activity, we performed in vitro cleavage assays using physiologically relevant magnesium‐like cations (Figure 4A,B). Notably, manganese ions elicited a pronounced catalytic effect on PS473 at both 1 mM and 10 µm. Kinetic analyses further showed that cleavage activity increased in a concentration‐dependent manner for both Mg^2^^+^ and Mn^2^^+^, with Mn^2^^+^ consistently supporting substantially higher catalytic efficiency, particularly under limiting metal‐ion conditions (Figure 4C,D). At a physiological‐like concentration of 0.5 mM, PS473 displayed an observed cleavage rate constant (*k*_obs_) of 0.1165 min^−^^1^ in the presence of Mn^2^^+^, which was more than twice the rate obtained with Mg^2^^+^ (*k*_obs_ = 0.0539 min^−^^1^) (Figure 4E,F). This disparity became even more pronounced at low metal‐ion concentrations: at 15.6 µM, the PS473 cleavage rate with Mn^2^^+^ reached 0.0154 min^−^^1^, exceeding the Mg^2^^+^ reaction rate (*k*_obs_ = 0.0009 min^−^^1^) by more than 17‐fold (Figure 4G,H).

These findings underscore the superior catalytic enhancement conferred by Mn^2^^+^, establishing it as a more potent and effective cofactor for PS473‐mediated RNA cleavage than Mg^2^^+^. This property highlights the potential of Mn^2^^+^‐based systems in amplifying ribozyme activity under physiologically relevant, metal‐ion‐limited conditions.

### 2.4 Construction and Characterization of the NKMOF‐101‐[Mn] Nanocarrier

**FIGURE 5:**
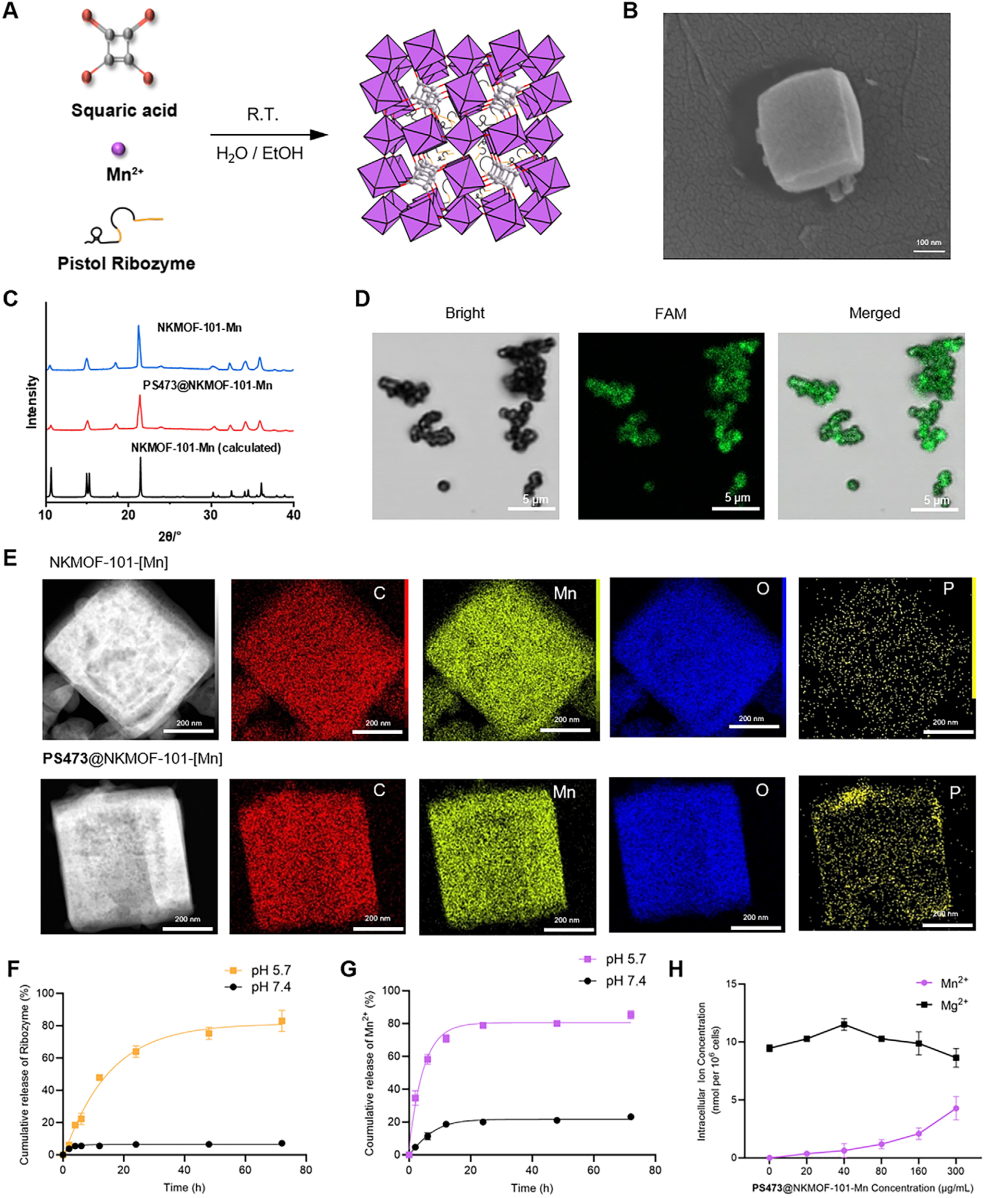
Synthesis, characterization, and functional validation of the Pistol ribozyme‐loaded MOF (PS473@NKMOF‐101‐[Mn]). (A) PS473@NKMOF‐101‐[Mn] was synthesized via a coordination reaction of squaric acid, Mn^2^^+^, and Pistol ribozyme in a mixed aqueous‒ethanol system at room temperature. (B) Scanning electron microscopy (SEM) image showing uniform polyhedral morphology of PS473@NKMOF‐101‐[Mn]. (C) Powder X‐ray diffraction (PXRD) patterns of NKMOF‐101‐[Mn] and PS473@NKMOF‐101‐[Mn] demonstrate preserved crystallinity consistent with the simulated reference. (D) Confocal laser scanning microscopy (CLSM) images confirming successful encapsulation of the FAM‐labeled Pistol ribozyme in NKMOF‐101‐[Mn], with clear colocalization in the bright‐field and fluorescence channels. (E) Transmission electron microscopy (TEM) and energy‐dispersive X‐ray spectroscopy (EDS) elemental mapping reveal distributions of C, Mn, O, and P in both NKMOF‐101‐[Mn] and PS473@NKMOF‐101‐[Mn]. (F, G) Cumulative release profiles showing more efficient release of both the ribozyme and Mn^2^^+^ under mildly acidic conditions (pH 5.7) than at neutral (pH 7.4), indicative of pH‐responsive behavior. (H) Intracellular metal ion quantification by LCP‐OES after 2 h of incubation with PS473@NKMOF‐101‐[Mn] reveals a dose‐dependent increase in the intracellular Mn^2^^+^ levels, whereas the Mg^2^^+^ concentration remains largely unchanged. All the data are shown as the mean (± SD), *n* = 3.

To address the dual challenges of limited catalytic efficiency and poor stability of ribozymes, we engineered a manganese‐based metal–organic framework (MOF), designated NKMOF‐101‐[Mn], that serves as a multifunctional nanocarrier capable of co‐delivering the ribozyme while simultaneously supplying Mn^2^^+^ ions to improve its catalytic performance. Unlike conventional Mn‐MOFs—typically synthesized through harsh solvothermal conditions that are incompatible with RNA loading, our platform is produced using a mild, aqueous, and room‐temperature one‐pot method. In this procedure, the ribozyme solution is first mixed with sodium squarate, followed by the addition of MnCl_2_ in a water–ethanol system, yielding PS473@NKMOF‐101‐[Mn] within 10 min (Figure 5A).

The resulting composite exhibited high crystallinity, as evidenced by scanning electron microscopy (SEM) and powder X‐ray diffraction (PXRD), which confirmed that ribozyme loading did not significantly disrupt the MOF's crystal structure (Figure 5B,C). Successful encapsulation of PS473 was verified by agarose gel electrophoresis and fluorescence imaging of FAM labeled ribozyme (Figure S2A,B; Figure 5D). SEM and TEM analyses further revealed uniform polyhedral nanoparticles of approximately 300 nm in diameter—an optimal size range for efficient endocytosis (Figure 5B,E). Elemental mapping by TEM–EDS demonstrated clear colocalization of phosphorus (derived from the ribozyme) with Mn within the framework (Figure 5E; Figure S3), indicating effective entrapment of the ribozyme within the nanoporous matrix. Dynamic light scattering (DLS) measurements confirmed that, within the test duration, NKMOF‐101‐[Mn] and PS473@NKMOF‐101‐[Mn] maintain particle sizes suitable for cellular internalization in both PBS and serum‐containing medium (Figure S2C). Changes in zeta potential among NKMOF‐101‐[Mn], PS473, and PS473@NKMOF‐101‐[Mn] (Figure S2D) further indicate electrostatic interactions between the MOF framework and the ribozyme. The encapsulation efficiency exceeded 80%, as determined by UV absorbance and electrophoresis (Figure S2E). Taken together, these results confirm that the ribozyme is efficiently and stably incorporated into the MOF carrier.

We next examined the release behavior of PS473@NKMOF‐101‐[Mn] under physiological and acidic conditions. As shown in Figure 5F, ribozyme release was minimal at neutral (pH 7.4) but was significantly accelerated under mildly acidic conditions (pH 5.7), exhibiting a sustained release profile over 48 h. Mn^2^^+^ release displayed a similar pH‐dependent trend, with markedly enhanced ion liberation under acidic conditions (Figure 5G). These findings indicate that the nanosystem enables coordinated release of both PS473 and Mn^2^^+^ in tumor‐mimicking acidic microenvironments, thereby supporting catalytic improvement and targeted delivery. Functionally, PS473@NKMOF‐101‐[Mn] retained robust catalytic activity even in the absence of externally supplied metal ions, attributable to the internally released Mn^2^^+^ (Figure S2F). Inductively coupled plasma optical emission spectroscopy (ICP–OES) further confirmed a concentration‐dependent increase in intracellular Mn^2+^ accumulation following treatment, whereas intracellular Mg^2^^+^ levels remained essentially unchanged (Figure 5H).

**FIGURE 6:**
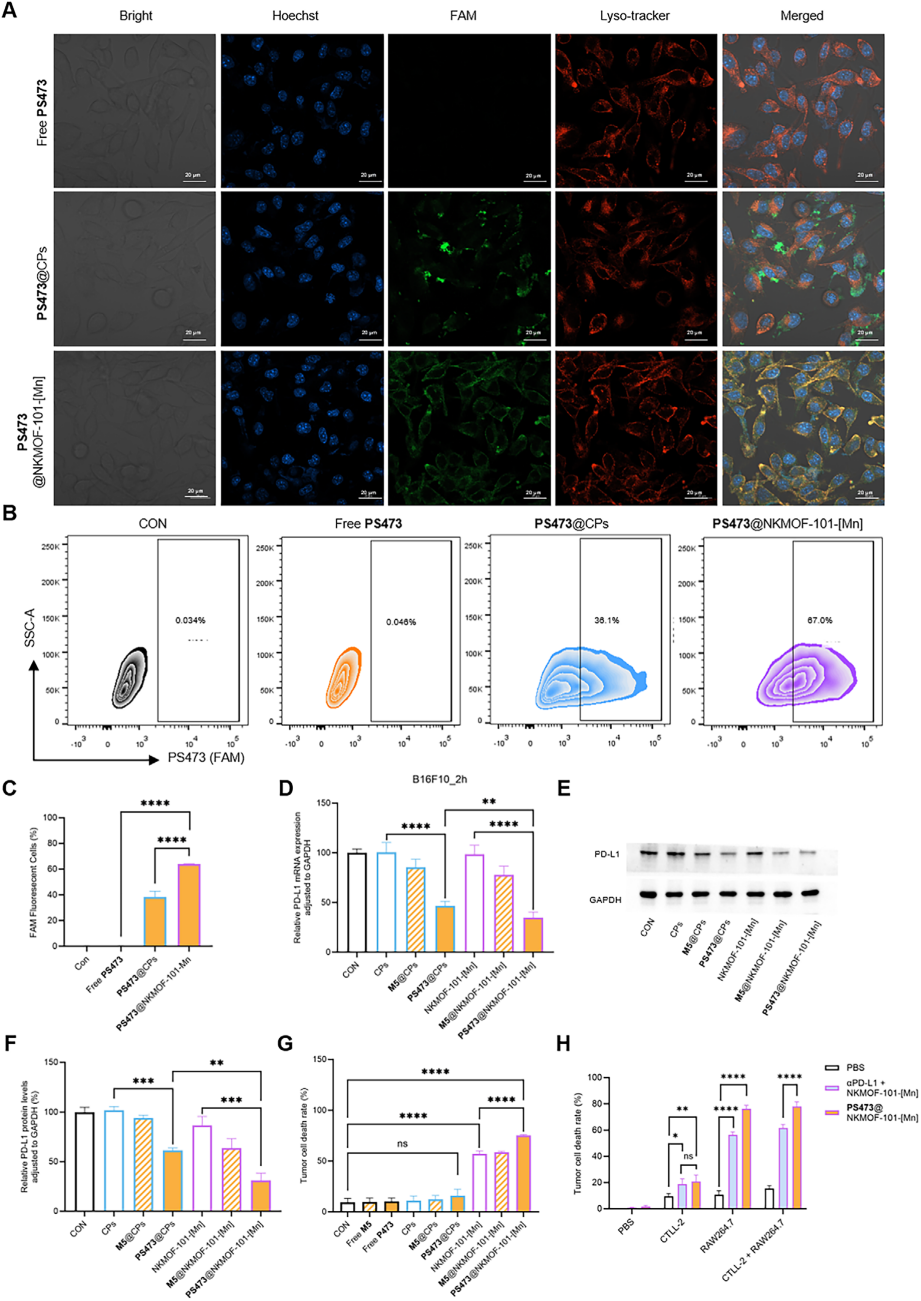
Intracellular delivery, PD‐L1 silencing, and immune‐mediated antitumor functions of PS473@NKMOF‐101‐[Mn]. (A) Confocal laser scanning microscopy (CLSM) images showing intracellular distribution of FAM‐labeled PS473 (Green) under different formulations. Scale bar = 20 µm. (B,C) Representative contour plots and quantitative flow‐cytometric analysis demonstrating enhanced cellular internalization, with PS473@NKMOF‐101‐[Mn] yielding the highest proportion of FAM‐positive cells (67.0%). (D) Quantitative PCR (qPCR) analysis of PD‐L1 mRNA levels across control group (CON), CPs‐transfected groups (CPs, M5@CPs, PS473@CPs), and MOF nanocarrier–transfected groups (NKMOF‐101‐[Mn], M5@NKMOF‐101‐[Mn], PS473@NKMOF‐101‐[Mn]) after 2 h in B16F10 cells. PS473@NKMOF‐101‐[Mn] produced the most substantial reduction in PD‐L1 expression. (E,F) Western blot analysis and densitometric quantification showing downregulation of PD‐L1 protein following 24 h of treatment with the indicated formulations. (G) Quantification of tumor‐cell killing efficiency by treated RAW264.7 macrophages, showing that MOF‐based formulations enhanced killing, with PS473@NKMOF‐101‐[Mn] exhibiting the highest activity. (H) Quantification of immune‐cell‐mediated tumor‐cell killing in co‐culture assays. CTLL‐2 T cells and/or NKMOF‐101‐[Mn]–activated RAW264.7 macrophages were co‐incubated with B16F10‐Luc cells under the indicated treatments. Both the PD‐L1 antibody+NKMOF‐101‐[Mn] and PS473@NKMOF‐101‐[Mn] enhanced immune‐cell cytotoxicity, yielding similarly high killing efficiency when combined with activated macrophages. All the data are presented as the mean (± SD), *n* = 3, with statistical significance indicated as ^**^ *p* < 0.01, ^***^ *p* < 0.001, and ^****^ *p* < 0.0001; ns = not significant.

Importantly, ribozyme stability assays demonstrated that PS473 encapsulated within NKMOF‐101‐[Mn] retained more than 50% of its catalytic activity after 12 h in serum and cell lysates, whereas the naked ribozyme was completely degraded within 2 h (Figure S2G,H). These results highlight the protective role of the MOF scaffold in preserving ribozyme integrity and prolonging its bioavailability. Cellular uptake was next evaluated by confocal microscopy and flow cytometry. Free PS473 exhibited minimal internalization, whereas PS473@NKMOF‐101‐[Mn] displayed robust intracellular fluorescence, exceeding that of the cationic polymer controls after 2 h (Figure 6A). Flow‐cytometric analysis further revealed a substantial increase in the fluorescence‐positive cells (67%) following treatment with PS473@NKMOF‐101‐[Mn] (Figure 6B,C; Figure S4C), accompanied by a sustained intracellular signal over time (Figure S4B). Together, these findings confirm the efficient cellular delivery and retention enabled by the MOF carrier.

In summary, NKMOF‐101‐[Mn] provides an integrated platform that enables ribozyme stabilization, Mn^2^^+^‐mediated catalytic enhancement, and pH‐responsive tumor‐targeted release. This multifunctional nanocarrier holds strong potential to advance the therapeutic application of RNA‐based tools in cancer immunotherapy.

### 2.5 In Vitro Inhibition of PD‐L1 Expression and Macrophage Activation

To evaluate the intracellular gene‐silencing activity of PS473@NKMOF‐101‐[Mn], we first determined a safe working concentration by CCK‐8 assay (Figure S4A). Both NKMOF‐101‐[Mn] and PS473@NKMOF‐101‐[Mn] showed dose‐dependent cytotoxicity, with minimal effects below 44 µg mL^−1^; therefore, 40 µg mL^−1^ was selected for subsequent studies. After 2 h incubation with PS473@NKMOF‐101‐[Mn], qPCR analysis revealed a rapid and significant reduction in PD‐L1 mRNA, exceeding all control formulations (Figure 6D). Western blot analysis at 24 h showed a similar trend, with PS473@NKMOF‐101‐[Mn] inducing markedly stronger PD‐L1 protein downregulation—approximately twice than that of the cationic polymer (Figure 6E,F).

The inactive M5@NKMOF‐101‐[Mn] mutant produced only modest suppression, confirming that catalytic activity is required for maximal gene silencing, although shared formulation‐related factors contribute to the minor residual knockdown. The superior performance of PS473@NKMOF‐101‐[Mn] reflects both efficient intracellular delivery and Mn^2^^+^‐mediated enhancement of ribozyme catalysis.

We next assessed the immunomodulatory activity of PS473@NKMOF‐101‐[Mn] using a macrophage‐mediated tumor‐killing assay. RAW264.7 macrophages were pretreated with the indicated formulations at 40 µg mL^−1^ (a non‐toxic dose confirmed by the CCK‐8 assay; Figure S4E) and co‐cultured with B16F10‐Luc cells (E:T = 4:1). Tumor viability was quantified by a luciferase‐based assay. As shown in Figure 6G, NKMOF‐101‐[Mn] alone activated macrophages to a moderate level (≈65% killing), markedly higher than free PS473 or PS473@CPs (≤25%). PS473@NKMOF‐101‐[Mn] achieved the strongest effect (≈80%), significantly surpassing all controls. The inactive M5@NKMOF‐101‐[Mn] group performed similarly to NKMOF‐101‐[Mn], indicating that the superior activity of PS473@NKMOF‐101‐[Mn] arises from dual contributions of catalytic PD‐L1 silencing and Mn^2^^+^‐driven macrophage activation.

To mechanistically explain the enhanced macrophage‐mediated tumor killing observed with PS473@NKMOF‐101‐[Mn], we profiled innate‐immune activation in RAW264.7 cells. PS473@NKMOF‐101‐[Mn] induced strong transcriptional upregulation of interferon‐stimulated genes (CXCL10, ISG15, ISG20) and pro‐inflammatory cytokines (IL‐6, TNF), together with elevated IFNB1, indicative of STING‐dependent type‐I interferon signaling (Figure S4D,F). Notably, PD‐L1 expression in macrophages was also significantly reduced (Figure S4D,F), relieving immune suppression within these innate effector cells. These data collectively confirm that PS473@NKMOF‐101‐[Mn] acts as both a gene‐silencing agent and a potent immune activator, providing a mechanistic basis for its superior macrophage‐mediated cytotoxicity.

To further confirm that PD‐L1 blockade underlies the immune‐cell‐mediated tumor killing, we introduced CTLL‐2 cells (murine T‐cell line) and a PD‐L1–neutralizing antibody as mechanistic controls. After pretreating cells with the indicated formulations at 40 µg mL^−1^ (a non‐toxic dose validated by the CCK‐8 assay; Figure S4G), CTLL‐2 cells alone exhibited only modest cytotoxicity toward B16F10‐Luc cells, whereas PD‐L1 antibody+NKMOF‐101‐[Mn] treatment or PS473@NKMOF‐101‐[Mn] both significantly enhanced T‐cell–mediated killing (Figure 6H). When combined with NKMOF‐101‐[Mn]–activated RAW264.7 macrophages, these two PD‐L1–targeting strategies yielded similarly high killing efficiencies (≈80–90%), indicating that relief of PD‐1/PD‐L1–mediated inhibition restores immune‐cell effector function and that PS473‐mediated PD‐L1 silencing effectively phenocopies pharmacological PD‐L1 blockade.

Together, these results demonstrate that PS473@NKMOF‐101‐[Mn] achieves two coordinated immunotherapeutic functions: efficient PD‐L1 ribozyme silencing and potent Mn^2^^+^‐driven activation of innate immunity. By simultaneously relieving immune suppression and boosting macrophage tumoricidal activity, the nanosystem provides a clear mechanistic basis for its superior antitumor efficacy.

### 2.6 In Vivo Antitumor Efficacy of PS473@NKMOF‐101‐[Mn] Nanotherapeutics

**FIGURE 7:**
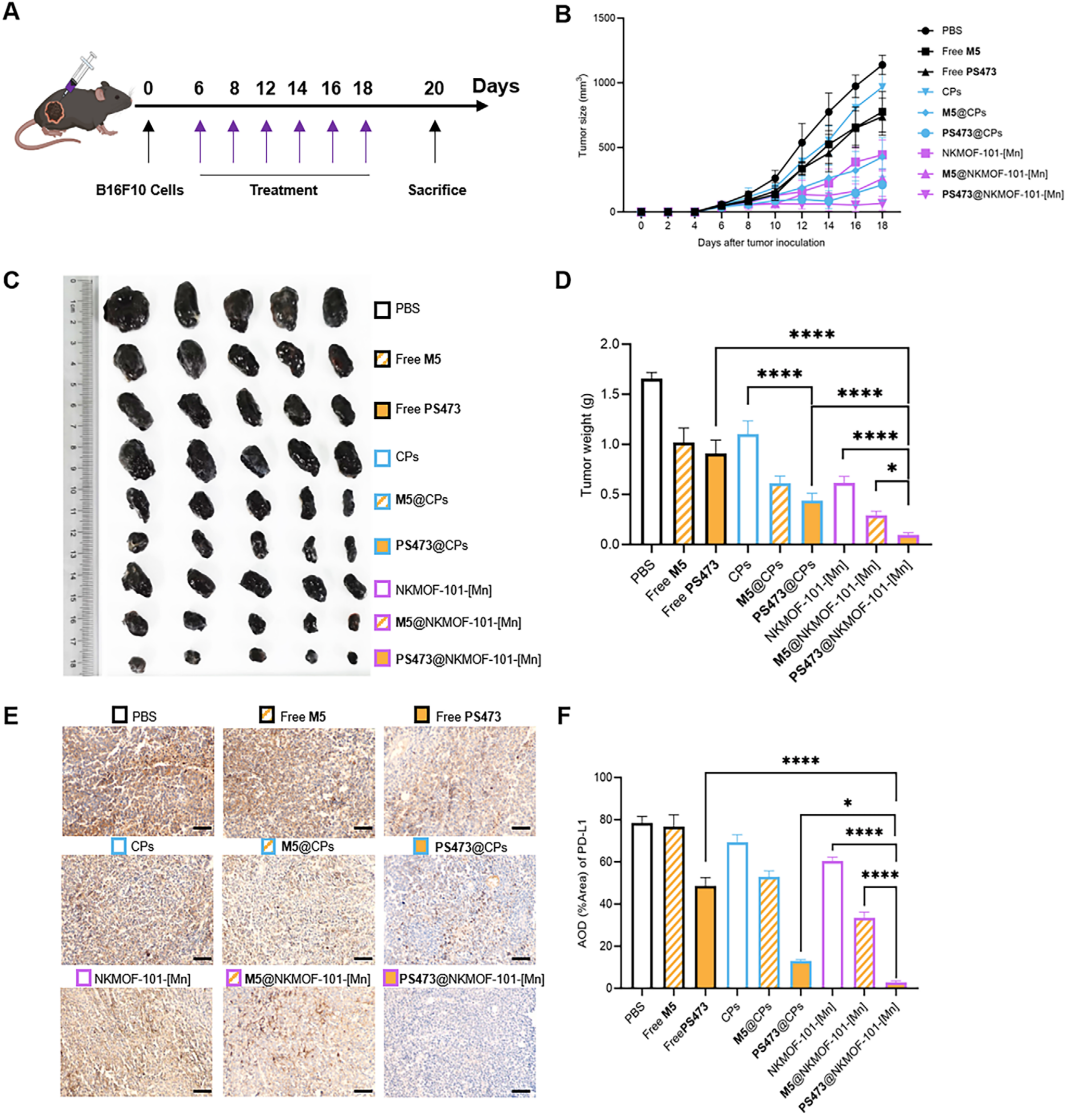
In vivo therapeutic efficacy of PS473@NKMOF‐101‐[Mn] in a B16F10 tumor‐bearing mouse model. (A) Schematic illustration of the treatment schedule. Tumor‐bearing mice received intratumoral injections of the indicated formulations on alternate days. (B) Tumor growth curves over the 18‐day treatment period. Black‐colored groups represent treatments without transfection reagents (PBS, Free M5, Free PS473), Cyan‐colored groups denote cationic polymer‐based formulations (CPs, M5@CPs, PS473@CPs). Purple‐colored groups indicate NKMOF‐101‐[Mn]–based formulations (NKMOF‐101‐[Mn], M5@NKMOF‐101‐[Mn], PS473@NKMOF‐101‐[Mn]; the same color scheme is used throughout). Among all treatments, PS473@NKMOF‐101‐[Mn] produced the most substantial inhibition of tumor growth. (C,D) Representative excised tumor images (C) and corresponding tumor weight quantification (D) further confirm the superior antitumor efficacy of PS473@NKMOF‐101‐[Mn] compared with the other controls. (E, F) Representative immunohistochemical staining (IHC) of PD‐L1 in tumor sections (brown) with quantification based on average optical density (AOD). Scale bar = 100 µm. PS473@NKMOF‐101‐[Mn] resulted in the most pronounced reduction in PD‐L1 expression among all groups. No mortality or treatment‐related clinical abnormalities were observed throughout the study. Data are presented as mean ± SD (*n* = 3). Statistical significance: ^*^ *p* < 0.05, ^**^ *p* < 0.01, ^***^ *p* < 0.001, and ^****^ *p* < 0.0001.

To assess the in vivo therapeutic potential of PS473@NKMOF‐101‐[Mn], we established a subcutaneous melanoma model by implanting B16F10 cells into C57BL/6 mice. Once tumors reached approximately 50 mm^3^, the mice were randomized into nine treatment groups (*n* = 5 per group): vehicle controls (PBS, CPs, NKMOF ‐ 101 ‐ [Mn]), active ribozyme formulations (Free PS473, PS473@CPs, PS473@NKMOF‐101‐[Mn]), and inactive mutant controls (Free M5, M5@CPs, M5@NKMOF‐101‐[Mn]). Each formulation was administered intratumorally every other day for 18 days, corresponding to a constant ribozyme dose of 20 µg per injection (Figure 7A, the dosage of materials is detailed in Section 4.10). Tumor growth curves (Figure 7B), representative tumor images (Figure 7C), and final tumor weights (Figure 7D) consistently demonstrated that PS473@NKMOF‐101‐[Mn] achieved the most pronounced therapeutic benefit. This group exhibited marked suppression of tumor progression, with an average inhibition rate of ≈91% compared with the PBS controls. Notably, this antitumor effect exceeded that of PS473@CPs (≈79.5%) and NKMOF‐101‐[Mn] alone (≈64.9%), underscoring the synergistic advantage of simultaneous ribozyme delivery and Mn^2^^+^‐mediated catalytic/immune activation.

Histopathological evaluation of tumor sections by H&E staining (Figure S5A) revealed marked cytological alterations in the PS473@NKMOF‐101‐[Mn] group, including cytoplasmic shrinkage, nuclear pyknosis, and substantial immune cell infiltration. These pathological features were considerably more pronounced than in any other treatment group, indicating enhanced tumor apoptosis and heightened antitumor immune activation. Consistent with these observations, immunohistochemical (IHC) staining demonstrated a significant reduction in PD‐L1 expression following PS473@NKMOF‐101‐[Mn] treatment (Figure 7E,F). This downregulation was further validated by Western blot and Quantitative PCR analysis of excised tumor tissues (Figure S5B–D), confirming the robust in vivo gene‐silencing effect of the Pistol ribozyme when delivered via Mn‐MOF nanosystem.

Collectively, these results demonstrate that PS473@NKMOF‐101‐[Mn] elicits potent antitumor activity by coupling effective PD‐L1 gene silencing with Mn^2^^+^‐driven immunomodulation, establishing this nanosystem as a promising platform for cancer immunotherapy.

### 2.7 Transcriptomic Profiling Reveals Immune Activation Mechanisms

**FIGURE 8:**
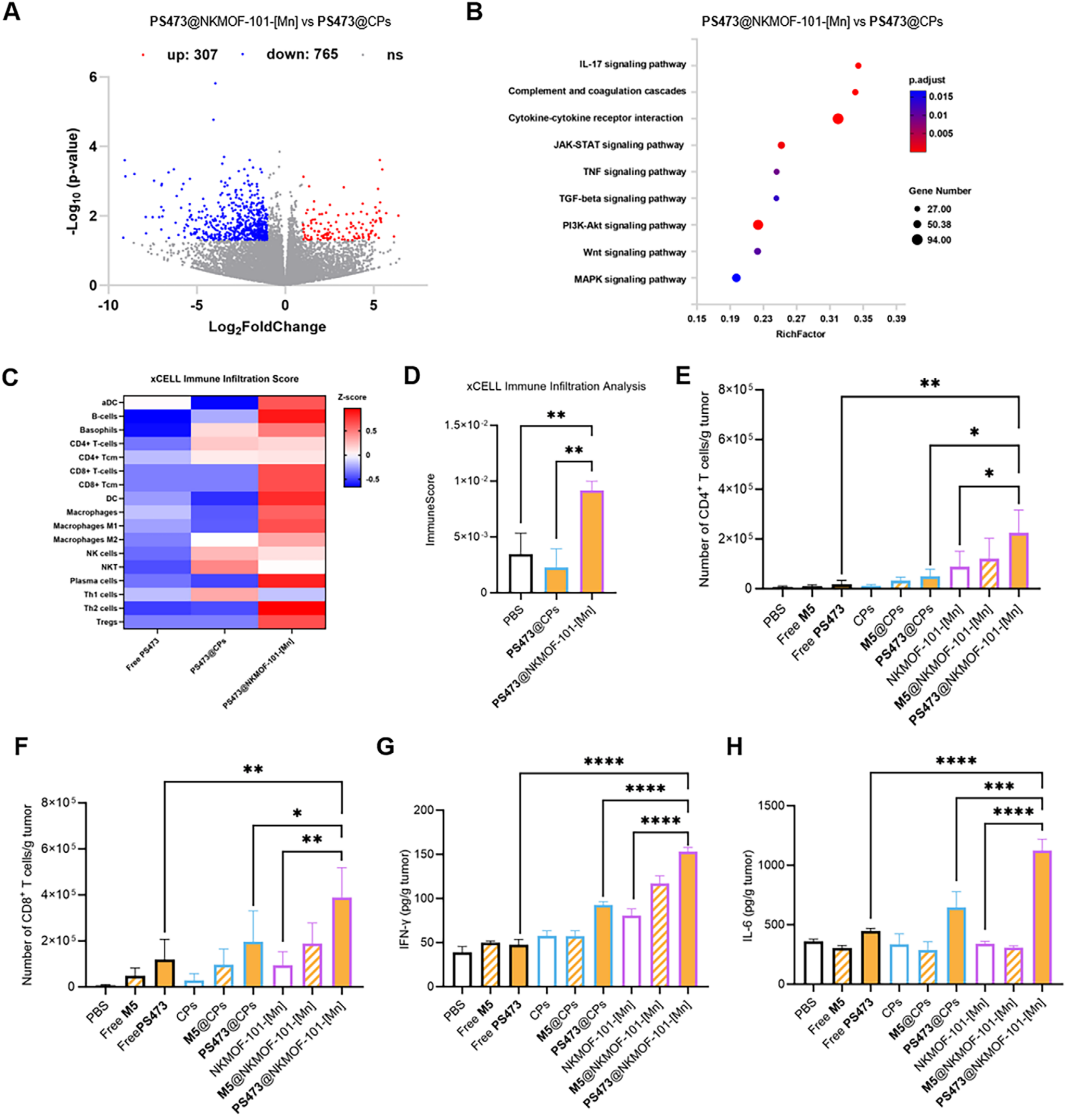
Transcriptome analysis and immune infiltration profiling following PS473‐mediated treatment. (A) Volcano plot comparing tumors treated with PS473@NKMOF‐101‐[Mn] and PS473@CPs, showing significantly upregulated genes (red), downregulated genes (blue), and nonsignificant genes (gray) (fold change > 2, adjusted *p* < 0.05). (B) KEGG pathway enrichment analysis of differentially expressed genes, with color intensity indicating the statistical significance of pathway enrichment. (C) Heatmap of xCell‐derived immune infiltration scores demonstrating increased infiltration of immune effector cells—including CD8^+^ T cells, CD4^+^ T cells, NK cells, and M1 macrophages—in the PS473@NKMOF‐101‐[Mn] group. (D) Quantitative comparison of the xCell immune infiltration scores confirming that PS473@NKMOF‐101‐[Mn] induces significantly stronger immune activation than does PS473@CPs or PBS. (E,F) Flow cytometry analysis of tumor‐infiltrating lymphocytes (TILs) reveals increased CD4^+^ T cells (E) and activated CD8^+^ T cells (F) populations following PS473@NKMOF‐101‐[Mn] treatment. (G, H) ELISA quantification of intratumoral cytokines showing robust induction of IFN‐γ (G) and IL‐6 (H)in the PS473@NKMOF‐101‐[Mn] relative to controls. Data are presented as mean ± SD (*n* = 3). Statistical significance: ^*^ *p* < 0.05, ^**^ *p* < 0.01, ^***^ *p* < 0.001, ^****^ *p* < 0.0001.

To elucidate the mechanistic basis of the potent antitumor and immunostimulatory effects of PS473@NKMOF‐101‐[Mn], we performed transcriptome‐wide RNA sequencing on excised tumor tissues. Compared with the cationic polymers (CPs) delivery system, PS473@NKMOF‐101‐[Mn] elicited a substantially broader transcriptomic response, with 307 genes significantly upregulated and 765 downregulated (Figure 8A). KEGG pathway enrichment analysis revealed strong activation of immune‐ and cell‐death–related signaling pathways, including cytokine–cytokine receptor interactions, TNF signaling, and the PI3K–Akt pathway (Figure 8B), collectively suggesting robust immunological engagement within the tumor microenvironment. Notably, PS473@NKMOF‐101‐[Mn] also triggered stronger immune‐related transcriptional changes than Free PS473 alone Figure S6A,B, underscoring the importance of Mn‐MOF–mediated delivery. To further validate these transcriptomic findings, we quantified immune cell infiltration using the xCell deconvolution algorithm [36]. PS473@NKMOF‐101‐[Mn] treatment markedly increased both the abundance and diversity of tumor‐infiltrating immune populations (Figure 8C), accompanied by significantly elevated composite immune scores (Figure 8D; Figure S6C). Together, these results demonstrate that the Mn‐based MOF platform substantially remodels the tumor immune microenvironment and enhances antitumor immune activation.

Flow cytometric analysis of tumor‐infiltrating lymphocytes further corroborated the transcriptomic findings (Figure S6D). PS473@NKMOF‐101‐[Mn] treatment resulted in a marked increase in CD4^+^ T‐cell infiltration—approximately fourfold higher than that observed in PBS controls (Figure 8E). Furthermore, A significant elevation in CD8^+^ cytotoxic T lymphocytes was also detected (Figure 8F), indicating enhanced recruitment and activation of effector T cells within the tumor. These immunological changes are consistent with restored T‐cell function following PD‐L1 silencing and support the conclusion that the nanosystem effectively reverses local immune suppression.

Cytokine profiling further supported the immunostimulatory effects of the treatment. Tumors treated with NKMOF‐101‐[Mn] alone presented elevated IFN‐γ levels (Figure 8G), consistent with Mn^2^^+^‐mediated activation of innate immune pathways. In parallel, the PS473‐containing formulations induced higher IL‐6 production (Figure 8H), reflecting enhanced immune cell engagement and inflammatory signaling within the tumor microenvironment.

Collectively, these data suggest that PS473@NKMOF‐101‐[Mn] amplifies antitumor immunity through coordinated mechanisms: Mn^2^^+^‐driven innate immune activation, increased immune‐cell infiltration, reversal of local immunosuppression, and strengthened T‐cell cytotoxic responses. These combined effects likely underlie the pronounced therapeutic efficacy observed in vivo.

### 2.8 Biosafety Evaluation

**FIGURE 9:**
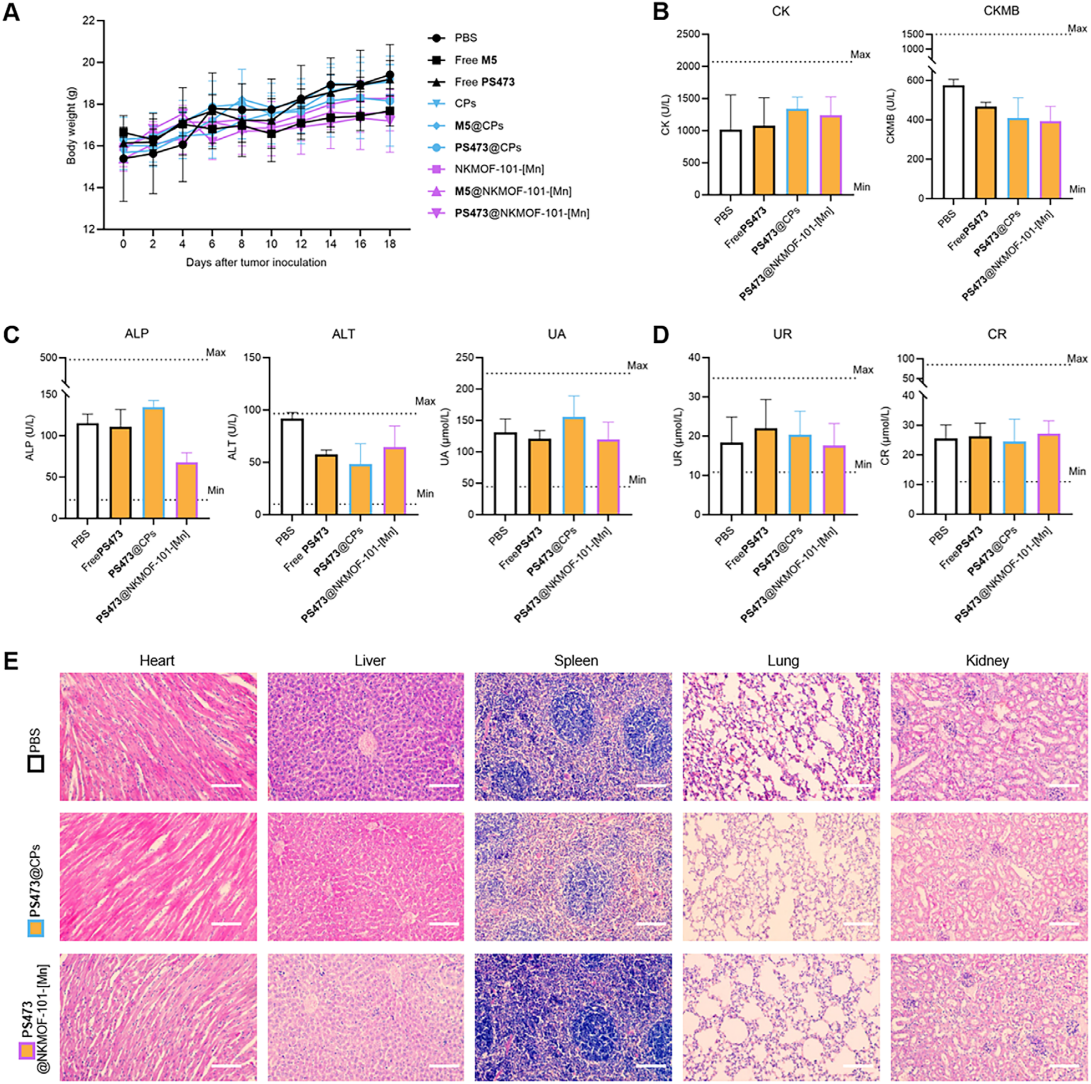
In vivo biosafety assessment of PS473@NKMOF‐101‐[Mn] in B16F10 tumor‐bearing mice. (A) Body weight monitoring throughout the treatment period showed no significant systemic toxicity in any group. (B) Serum levels of cardiac biomarkers, including creatine kinase (CK) and creatine kinase‐MB (CKMB), remained within normal ranges, indicating minimal cardiotoxicity. (C) Liver function markers—alkaline phosphatase (ALP), alanine transaminase (ALT), and uric acid (UA)—showed no abnormal elevation across treatment groups. (D) Kidney function indicators, including urea (UR) and creatinine (CR), likewise showed no significant differences, suggesting preserved renal function. (E) Representative H&E‐stained histological sections of the heart, liver, spleen, lung, and kidney revealed no obvious signs of inflammation, necrosis, or structural abnormalities in the PS473@NKMOF‐101‐[Mn] group compared with the PBS or polymer control groups. Scale bar = 100 µm.

Biocompatibility is a critical determinant of the translational potential of nanotherapeutics. To assess the in vivo safety of PS473@NKMOF‐101‐[Mn], we monitored body weight and examined major organs for histopathological alterations. Throughout the treatment period, all mice exhibited steady weight gain with no observable signs of acute toxicity or abnormal behavior (Figure 9A). Serum biochemical analysis demonstrated that all eight evaluated parameters—including key hepatic and renal function markers—remained within normal physiological limits (Figure 9B–D), suggesting no detectable disruption of systemic metabolism or major organ function. Consistently, histological assessment of the heart, liver, spleen, lung, and kidney using H&E staining revealed preserved tissue architecture, with no signs of inflammation, necrosis, or other pathological changes across all treatment groups (Figure 9E; Figure S7).

These findings collectively demonstrate the excellent biocompatibility of the PS473 ribozyme, the NKMOF‐101‐[Mn] nanocarrier, and the assembled nanosystem. Importantly, the data indicate that the therapeutic regimen does not induce measurable systemic toxicity or irreversible organ damage at the administered doses, supporting its safety profile and suitability for further translational development. We note that core body temperature was not recorded in this study; although no behavioral or biochemical abnormalities indicative of systemic inflammation were observed, temperature monitoring will be incorporated in future work to further strengthen the safety assessment.

## 3 Discussion

The therapeutic targeting of immune checkpoints, particularly the PD‐1/PD‐L1 axis, has reshaped the landscape of cancer immunotherapy. However, clinical limitations—including variable patient response, immune resistance, and suboptimal efficacy in noninflamed tumors—continue to hinder broader success. Here, we present a dual‐function therapeutic nanosystem, PS473@NKMOF‐101‐[Mn], that synergizes Pistol ribozyme–driven checkpoint gene silencing with Mn^2+^‐based innate immune stimulation via a biocompatible MOF nanocarrier. This strategy exemplifies how catalytically active RNA molecules can be reengineered for precise gene regulation in cancer immunotherapy while preserving the structural and chemical logic inherent to ribozyme biology.

This study represents a meaningful extension of the ribozyme field, where most previous work has focused on in vitro enzymology or structural elucidation. Building upon prior investigations into ribozyme catalytic mechanisms and GU site cleavage specificity, we have adapted the Pistol ribozyme—a naturally occurring RNA catalyst—for cellular and in vivo application against a toumor progression‐associated transcript (PD‐L1). By combining rational design with in vitro kinetic assays and cellular PD‐L1 knockdown as functional readouts, we identified a highly active and selective ribozyme variant, PS473, which exhibited robust site‐specific cleavage under quasiphysiological conditions and potent checkpoint silencing in tumor cells. The use of a Mn^2^^+^‐responsive MOF as both a delivery vehicle and a catalytic cofactor source helps to overcome the long‐standing bottlenecks of ribozyme instability and poor cellular uptake without altering the intrinsic catalytic framework of the ribozyme.

This work leverages the unique properties of a mildly synthesized Mn‐based MOF (NKMOF‐101‐[Mn]) to enable ribozyme stabilization and metal‐ion codelivery in a tumor‐adaptive manner. The MOF synthesis route, which avoids harsh solvothermal conditions, was designed specifically to accommodate structurally fragile RNA molecules, illustrating how material chemistry can be tailored to the biological constraints of catalytic RNAs. From an MOF research standpoint, this platform highlights a new biomedical use case: dynamic corelease of RNA therapeutics and immune‐active metal ions to orchestrate spatially resolved, synergistic therapeutic effects.

Mechanistically, our kinetic analyses demonstrate that Mn^2^^+^ markedly improves PS473‐mediated RNA cleavage compared with Mg^2^^+^, particularly under conditions of limited metal‐ion availability, with more than a 17‐fold increase in the observed rate at low micromolar concentrations. This behavior is consistent with previous biochemical and computational studies showing that Pistol ribozymes display higher catalytic activity in Mn^2^^+^ than in Mg^2^^+^, likely reflecting the ability of Mn^2^^+^ to act as a stronger Lewis acid and to more efficiently activate coordinated water molecules within the established general acid–base catalytic framework. Although the precise atomistic details were not dissected here, our data support a model in which Mn^2^^+^ serves as a more effective catalytic cofactor for Pistol ribozymes under physiologically relevant metal‐ion conditions.

Control experiments with the catalytically inactive mutant M5 and a PD‐L1‐blocking antibody further clarify the mechanism of checkpoint modulation. M5, which retains the same substrate‐binding arms but lacks catalytic activity, produced only partial PD‐L1 reduction in cells and in tumor tissue, consistent with non‐catalytic effects such as RNA–RNA hybridization–induced translational interference or engagement of endogenous RNA‐decay pathways. In contrast, PS473 consistently achieved deeper suppression of PD‐L1 at both the mRNA and protein levels, supporting a dominant contribution from bona fide ribozyme‐mediated cleavage rather than binding alone. Moreover, PD‐L1 monoclonal antibody controls elicited similar enhancements in T‐cell‐mediated tumor cell killing, reinforcing that PD‐L1 checkpoint blockade is a central mechanism of the observed antitumor responses and that PS473@NKMOF‐101‐[Mn] functions as a ribozyme‐based analogue of immune checkpoint inhibition.

From the perspective of RNA therapeutics, this study offers a generalizable framework for expanding ribozyme‐based interventions beyond structural or mechanistic studies. The ability to rationally select target sites, increase catalytic rates with physiologically relevant cofactors, and ensure intracellular bioavailability opens new possibilities for applying ribozymes to other oncogenic transcripts, immune checkpoints, or noncoding RNAs. Compared with antisense oligonucleotide or siRNA approaches, ribozymes offer rapid, site‐specific, and catalytically amplifying gene regulation and may offer a lower risk of off‐target effects owing to their dual dependence on sequence complementarity and the 3D folding of the substrate.

In conclusion, this work demonstrates that the catalytic capacity of ribozymes can be productively integrated with the structural intelligence of MOFs to achieve localized, durable, and multimodal cancer immunotherapy. PS473@NKMOF‐101‐[Mn] thus offers a proof‐of‐concept for modular ribozyme nanotherapeutics, bridging fundamental RNA catalysis and translational tumor biology. By simultaneously addressing the delivery and activation bottlenecks of ribozyme‐based drugs, our approach lays a foundation for further exploration of catalytic RNA as a programmable class of precision medicines.

## 4 Materials and Methods

### 4.1 Preparation of Pistol Ribozyme and PDL1 mRNA

The Pistol ribozyme was designed on the basis of its crystal structure (PDB ID: 5K7C [16]), which exhibits site‐specific cleavage activity at GU dinucleotides within RNA. The coding sequence (CDS) of the PD‐L1 gene (NM_021893.3) was analyzed, and its mRNA secondary structure was predicted to identify accessible, unpaired GU sites as potential ribozyme targets. DNA primers containing T7 RNA polymerase promoter sequences (synthesized by GENEWIZ) were used for in vitro transcription. The PD‐L1 CDS was amplified via PCR using the pCMV‐PD‐L1 plasmid (obtained from Mailgene Biosciences Co., Ltd., Beijing, China) as a template. Both the Pistol ribozyme and PD‐L1 RNA substrates were transcribed in vitro via a laboratory‐established transcription system. The resulting RNA products were purified via 8 M urea polyacrylamide gel electrophoresis (urea‐PAGE), quantified via a NanoDrop spectrophotometer, and stored at −20°C. We mutated the bases in the catalytic center of the Pistol ribozyme (i.e., G40‐U and C41‐A) with reference to Biochemical analysis of pistol self‐cleaving ribozymes [22, 37], yielding the ideal mutant ribozyme M5 that lacks catalytic cleavage activity.

### 4.2 In Vitro Ribozyme Cleavage Assay

Ribozyme and RNA substrates were annealed by heating at 65°C for 10 min, followed by gradual cooling to room temperature. The cleavage reaction was carried out in buffer containing 5 mM HEPES and 20 mM NaCl, with 1 µM RNA substrate and 1 µM ribozyme. Metal chloride salts were added as required to a final concentration of 1 mM. The reaction mixtures were incubated at 37°C for the indicated time periods and then quenched by adding an equal volume of termination buffer (90% formamide, 50 mM EDTA, 0.05% xylene cyanol, 0.05% bromophenol blue). The cleavage products were resolved via 10–20% denaturing urea‐PAGE, stained with GelRed, and visualized via a PXI9 imaging system (SYNGENE). Band intensities were quantified via ImageJ, and apparent cleavage rate constants (*k*_obs_) were determined by fitting the data to a single‐exponential decay model [38].

### 4.3 Assembly and Ribozyme Release Profiling of PS473@NKMOF‐101‐[Mn]

Squaric sodium was synthesized by dissolving squaric acid (182 mg, 1.6 mmol) and NaOH (128 mg, 3.2 mmol) in 16 mL of water. The solution was partially evaporated at room temperature to remove approximately 80% of the water, leading to the formation of squaric sodium microcrystals [33]. To prepare PS473@NKMOF‐101‐[Mn], an aqueous solution of squaric sodium (22.1 mg mL^−1^, 0.14 M, 1.0 mL) was mixed with 200 µL of PS473 ribozyme (200 µM). A manganese(II) chloride tetrahydrate solution (27.7 mg mL^−1^, 0.14 M, 1.0 mL) was prepared in a water–ethanol mixture (25–50% ethanol). The two solutions were combined and incubated at room temperature for 10 min. The resulting nanoparticles were collected by centrifugation at 8000 rpm for 3 min and washed with excess water. To verify ribozyme encapsulation, the FAM‐labeled PS473 ribozyme was loaded into NKMOF‐101‐[Mn] via the same one‐step procedure. The incorporation of the ribozyme was confirmed via fluorescence imaging via confocal microscopy. To evaluate ribozyme release, 1 mg of PS473@NKMOF‐101‐[Mn] was divided into three aliquots and incubated at 37°C in buffers of pH 7.4, pH 5.7, or with 100 mM EDTA. At predetermined time points, 5 µL of the supernatant was collected, mixed with loading buffer, and analyzed via 2% agarose gel electrophoresis. The gel images were analyzed via ImageJ to quantify ribozyme release.

### 4.4 Stability Evaluation of Ribozymes in Biological Media

To assess the protective effect of MOF encapsulation on ribozyme stability, 20 µL reaction mixtures containing 20 µM ribozyme were prepared. RNase A (5 mg mL^−1^), 10% freshly isolated mouse serum, or cell lysate was added to simulate degradation conditions. PS473@NKMOF‐101‐[Mn] was included at a concentration of 50 mg mL^−1^. The mixtures were incubated at 37°C for 2 ‐ 24 h. Following incubation, the samples were analyzed via 2% agarose gel electrophoresis. Gel images were captured via a UV302 imaging system and quantified via ImageJ software.

### 4.5 Confocal Imaging of Cellular Uptake and Localization

B16F10 cells were seeded in confocal imaging plates at a density of 2 × 10^5^ cells per well and incubated overnight. After coincubation with FAM‐labeled PS473 for 2, 6, or 12 h, the cells were stained with LysoTracker Red (Beyotime) and Hoechst 33342 (Beyotime). The cellular uptake and localization of the ribozyme‐MOF complexes were observed via a laser scanning confocal microscope (LSM 800 with Airyscan).

### 4.6 pH‐Responsive Mn^2^^+^ Release and Cellular Uptake Analysis

We prepared buffer solutions at pH 5.7 and pH 7.4 (5 mM HEPES and 20 mM NaCl), placed 5 mg mL^−1^ of the material in 500 µL of each solution, collected 5 µL samples at different time points, diluted the samples to 5 mL in the same solution, and stored them at −20°C for later analysis. Detection was performed via LCP‐OES (inductively coupled plasma‒optical emission spectrometry). For cell sample preparation, cells were treated with different concentrations of PS473@NKMOF‐101‐[Mn] at a density of 5 × 10^5^ cells in 12‐well plates for 2 h, collected, lysed by sonication, and then analyzed for Mn^2^^+^ and Mg^2^^+^ concentrations via LCP‐OES.

### 4.7 Gene Silencing Assay Using PS473 in Tumor Cells

Mouse melanoma (B16F10) and breast cancer (4T1) cells were cultured in RPMI 1640 and DMEM, respectively, at 37°C in a humidified atmosphere containing 5% CO_2_. The culture media were supplemented with 10% fetal bovine serum (FBS), 1% penicillin (100 U mL^−1^), and 1% streptomycin (100 µg mL^−1^). The cells were seeded at 2 × 10^5^ cells per well in 12‐well plates and incubated for 12 h to allow adhesion. The PS473 ribozyme was delivered into cells via either the cationic polymer HighGene transfection reagent (RM09014; ABclonal Biotechnology Co., Ltd.) or the NKMOF‐101‐[Mn] nanocarrier. After appropriate incubation, total RNA was extracted via TRIzol reagent for quantitative PCR (qPCR), and total protein was extracted via RIPA buffer for Western blot (WB) analysis. GAPDH was used as an internal control for both qPCR and WB. The qPCR primers used were as follows: PD‐L1: forward 5′‐ATGAGGATATTTGCTGGCATT‐3′, reverse 5′‐TTACGTCTCCTCGAATTGTG‐3′; GAPDH: forward 5′‐ACCCAGAAGACTGTGGATGG‐3′, reverse 5′‐TTCAGCTCAGGGATGACCTT‐3′. All DNA strands and primers were chemically synthesized by GENEWIZ and Tsingke Biotechnology Co., Ltd. (China). The primary antibodies used included an anti‐PD‐L1 mouse monoclonal antibody (dilution ratio 1:10000, Proteintech 66248‐1) and a GAPDH mouse monoclonal antibody (dilution ratio 1:20000, Proteintech 60004−1). The secondary antibody used was HRP‐conjugated goat antimouse IgG (dilution ratio 1:10000, Proteintech SA00001−1).

### 4.8 Cytotoxicity Assay

B16F10, CTLL‐2, and RAW264.7 cells (1 × 10^4^ cells per well) were seeded in 96‐well plates and incubated overnight at 37 °C in a humidified 5% CO_2_ atmosphere to allow cell attachment. Various concentrations of NKMOF‐101‐[Mn] or PS473@NKMOF‐101‐[Mn] were then added and incubated with the cells for 24 h. Following treatment, the culture medium was replaced with 10% CCK‐8 solution (v/v in complete medium), and the cells were further incubated for 2 h at 37°C. The absorbance at 450 nm was measured via a microplate reader (Infinite F50, TECAN) to assess cell viability.

### 4.9 In Vitro Immune Cell–Mediated Tumor Killing Assay

To further evaluate the immunomodulatory potential of PS473@NKMOF‐101‐[Mn], we assessed macrophage‐mediated cytotoxicity against tumor cells. B16F10‐LUC cells were first seeded in 12‐well plates, and then RAW264.7 cells or CTLL‐2 cells were added at an effector‐to‐target (E:T) ratio of 4:1. Subsequently, the cocultures were treated with PBS, anti–PD‐L1 antibody (0.1 µg mL^−^^1^, 10F.9G2, TargetMol) combined with NKMOF‐101‐[Mn] (40 µg mL^−^^1^), or PS473@NKMOF‐101‐[Mn] (40 µg mL^−^^1^) for 24 h. Then, the cells were lysed to release luciferase, and the luciferase activity was detected using the Firefly Luciferase Reporter Gene Cell Lysate Detection Kit (RG005, Beyotime) with a microplate reader (Spark) [33].

### 4.10 In Vivo Antitumor Study

B16F10 melanoma cells (1 × 10^6^ cells in 50 µL of PBS) were subcutaneously injected into the right flank of female C57BL/6 mice. On day 6, when the tumor volume reached approximately 50 mm^3^, the mice were randomly divided into nine groups and treated every two days via intratumoral injection with one of the following formulations: PBS, Free M5, Free PS473, CPs, M5@CPs, PS473@CPs, NKMOF‐101‐[Mn], M5@NKMOF‐101‐[Mn], or PS473@NKMOF‐101‐[Mn]. Each treatment group received 20 µg of ribozyme per dose. Based on nucleic‐acid loading and encapsulation efficiency measurements, the nucleic‐acid‐to‐material mass ratio in MOF‐based formulations was approximately 1:4. Accordingly, each intratumoral dose of 80 µg PS473@NKMOF‐101‐[Mn] delivered: ≈20 µg ribozyme (1 mg kg^−^^1^), ≈15 µg Mn^2^^+^ (1 mg kg^−^^1^), ≈33 µg squaric acid ligand (1.65 mg kg^−^^1^), and ≈12 µg bound water. This material dose (≈4 mg kg^−^^1^) is below the ranges commonly used in Mn‐MOF–based cancer immunotherapy studies [39], and the corresponding Mn^2^^+^ exposure falls within the reported safety limits for localized intratumoral administration. Tumor volumes and body weights were recorded every two days throughout the treatment period. After 14 days, the tumors were excised and weighed to evaluate therapeutic efficacy. The tumor inhibition rate (TIR) was calculated as:TIR = (1 − Mean Tumor Weight of Treatment Group / Mean Tumor Weight of PBS Group) × 100%. All animal procedures were approved by the Animal Ethics Committee of Nankai University (Approval No. TJUE‐2023‐081).

### 4.11 Immune Cell Profiling in Tumor Tissues

Tumor tissues were harvested from B16F10 tumor‐bearing mice 48 h after the final treatment. Single‐cell suspensions were prepared from the excised tumors, followed by incubation with 1% FBS in PBS for 3 min. The cells were then centrifuged and fixed with 4% paraformaldehyde. For immune cell analysis, the cells were stained with the following flow cytometry antibodies: PE‐conjugated anti‐CD3, APC‐conjugated anti‐CD4, and FITC‐conjugated anti‐CD8 (all purchased from Chengdu Zen‐Bioscience Co., Ltd.). The immune cell populations were quantified via a BD LSRFortessa flow cytometer. Additionally, the levels of interferon‐gamma (IFN‐γ) and interleukin‐6 (IL‐6) in tumor homogenates were measured via ELISA kits (Wuhan Servicebio Technology Co., Ltd.).

### 4.12 Expression Analysis in Tumor Tissues

Tumor tissues were collected, fixed in 4% paraformaldehyde, dehydrated, and embedded in paraffin. Tissue sections (4 µm thick) were deparaffinized, subjected to antigen retrieval via citrate buffer (pH 6.0), and stained via a two‐step immunohistochemistry (IHC) kit (Elabscience Biotechnology Co., Ltd.) with appropriate primary and secondary antibodies. PD‐L1 expression levels were visualized and quantified via ImageJ software. For protein‐level analysis, total protein was extracted from tumor tissues via RIPA lysis buffer, and PD‐L1 expression was evaluated via Western blotting.

### 4.13 Biosafety Evaluation

The body weights of the experimental mice were monitored every two days throughout the treatment period. Following the final treatment, blood samples were collected for biochemical analysis. Major organs, including the heart, liver, spleen, lungs, and kidneys, were harvested and subjected to hematoxylin–eosin (H&E) staining via a commercial HE staining kit (Beijing Solarbio Science & Technology Co., Ltd.). Histopathological examination was performed to assess potential tissue damage and evaluate the overall biosafety of the treatment.

### 4.14 Statistical Analysis

All the data were shown as the mean ± standard deviation (SD) and were analyzed with GraphPad Prism 10 software. Statistical differences were analyzed via two‐tailed unpaired Student's *t* test or one‐way ANOVA, and ^*^ *p*<0.05, ^**^ *p*<0.01, ^***^ *p*<0.001, and ^****^ *p*<0.0001 were considered statistically significant.

## Author Contributions

M.Z., S.Q., Y.L., and Y.C. supervised the study. M.Z. and S.Q. designed the study and experiments. M.Z. and S.Q. performed the experiments. Z.M. and J.B. conducted the statistical analysis of these results. M.Z. and S.Q. wrote the draft manuscript. J.Z., Z.X., Z.L., X.Z., and X.Y. discussed the results and edited the manuscript. M.Z. and Y.J. reviewed and revised the manuscript.

## Funding

This work was supported by the National Key Research and Development Program of China (2023YFA0913800), the National Natural Science Foundation of China (22371136, 21877065, 82111530210, 32360709), the Key Project of Tianjin Municipal Natural Science Foundation of China (24ZXZSSS00020, 25ZXZSSS00090), the Haihe Laboratory of Synthetic Biology (22HHSWSS00008), the Frontiers Science Center for New Organic Matter, Nankai University (63181206), and the Fundamental Research Funds for the Central Universities, Nankai University (035‐63191735, 035–63221328).

## Conflicts of Interest

The authors declare no conflicts of interest.

## Supporting information




**Supporting File**: advs74214‐sup‐0001‐SuppMat.docx.

## Data Availability

All data needed to evaluate the conclusions in the paper are present in the paper and/or the Supplementary Materials. The raw RNA sequencing data of tumor tissues have been deposited in the Gene Expression Omnibus (GEO) database under the accession number GSE302201, which can be accessed via the following link: www.ncbi.nlm.nih.gov/geo/query/acc.cgi?acc=GSE302201.
