# Supplementary material for: Synergistic Mn‐MOF Activation of Pistol Ribozymes for Cancer Immunotherapy
Source: Adv Sci (Weinh). 2026 Feb 10;13(20):e17912. doi: 10.1002/advs.202517912 (PMC13067846; doi:10.1002/advs.202517912)
Supplement: Supplementary file 1 — Supporting File: advs74214‐sup‐0001‐SuppMat.docx. [file ADVS-13-e17912-s001.docx]

**Synergistic Mn-MOF Activation of Pistol Ribozymes for Cancer Immunotherapy**

*Ming Zhao et al.*

*Corresponding author. Email: yvliu@nankai.edu.cn

Supplementary Materials

**This PDF file includes:**

Supplementary Text: The Catalytic Mechanism of the Pistol Ribozyme and Design Strategies for Its Variants.

Figs. S1 to S7

Tables S1 to S4

References

**The catalytic mechanism of the Pistol ribozyme**

Small self-cleaving ribozymes, including the Pistol ribozyme, catalyze RNA strand scission through a 2′-O-transphosphorylation reaction that relies on a coordinated network of general acid–base catalysis. In this mechanism, four classical catalytic strategies—(α) in-line alignment of the 2′-OH nucleophile, (β) stabilization of the dianionic transition state, (γ) general base activation of the 2′-OH, and (δ) general acid protonation of the 5′-oxygen leaving group—operate simultaneously within a compact RNA active site. Crystallographic analysis of a deactivated Pistol ribozyme, in which the 2′-OH nucleophile was replaced by deoxyribose, revealed that a conserved guanine (G40) is positioned proximal to the 2′-position that would serve as the nucleophile in the active ribozyme.^[1]^ This observation led to the initial proposal that G40 could function as the general base. However, subsequent biochemical studies have challenged this assignment. In particular, substitution of G40 with 2-aminopurine resulted in slightly enhanced catalytic activity, inconsistent with the expected loss-of-function phenotype for a general base residue.^[2]^ Moreover, G40 does not conform to the conserved L-platform/L-scaffold architecture shared by G+M ribozymes.^[3]^ In contrast, another conserved guanine, G42, exhibits sensitivity to 2-aminopurine substitution and is compatible with the L-platform/L-scaffold framework, supporting its role as the general base in the primary catalytic pathway.^[2, 3]^ In this model, G42 abstracts a proton from the 2′-OH nucleophile, while a Mg²⁺-coordinated water molecule donates a proton to the leaving group, consistent with a general acid–base catalytic mechanism.

Importantly, although Mg²⁺ is the most common physiological cofactor, many other divalent cations—such as Mn²⁺, Ca²⁺, Co²⁺, and Cd²⁺—can substitute for Mg²⁺ and support catalysis because they maintain similar inner-sphere coordination chemistry and can activate bound water molecules as proton donors.^[4, 5]^ Among these, Mn²⁺ is particularly effective, owing to its higher Lewis acidity and stronger activation of coordinated water, allowing it to fulfill the general-acid role even more efficiently than Mg²⁺ in certain ribozymes.^[3]^ This property provides a mechanistic rationale for the use of Mn-MOF nanomaterials in this study: Mn²⁺ released from the MOF framework enhances the acid–base catalytic environment of the Pistol ribozyme and strengthens its cleavage efficiency.

Complementary computational studies have further clarified proton-transfer pathways involving G40, the catalytic metal ion, and its coordinated water ligands, supporting a unified model in which a conserved guanine acts as the general base, a divalent metal–bound water functions as the general acid, and the metal ion stabilizes the transition state.^[6]^

**Design of Pistol Ribozyme Variants**

To evaluate the effectiveness of Pistol ribozymes in targeting PD-L1 mRNA, we designed a series of trans-acting ribozymes that selectively cleave RNA substrates at GU dinucleotides—the preferred scissile motifs for Pistol ribozymes. The coding sequence (CDS) of mouse PD-L1 mRNA (NM_021893.3) was scanned to identify all GU dinucleotides that could serve as potential cleavage sites. Based on this mapping, we engineered a library of Pistol ribozyme variants using the first reported crystal structure of the Pistol ribozyme (PDB: 5K7C ^[7]^) as the structural template—hereafter referred to as “Pistols”—to target individual GU sites across the PD-L1 CDS.

Each Pistol ribozyme was named according to the nucleotide position of its intended cleavage site within the PD-L1 CDS. For example, the ribozyme designed to cleave after the GU motif at position 473 was designated PS473. Variants with modified P3 stem lengths were further annotated by adding the number of base pairs after a hyphen (e.g., PS473-16), as the P3 stem length is a key determinant of substrate recognition and target specificity.

An inactive Pistol ribozyme mutant (**M5**) was also generated by mutating the conserved G40 and G41 nucleotides to UA, following the strategy reported by Breaker and colleagues in RNA 21(11):1852–1858,^[8]^ which demonstrated that these substitutions abolish the catalytic activity of the Pistol ribozyme.

**
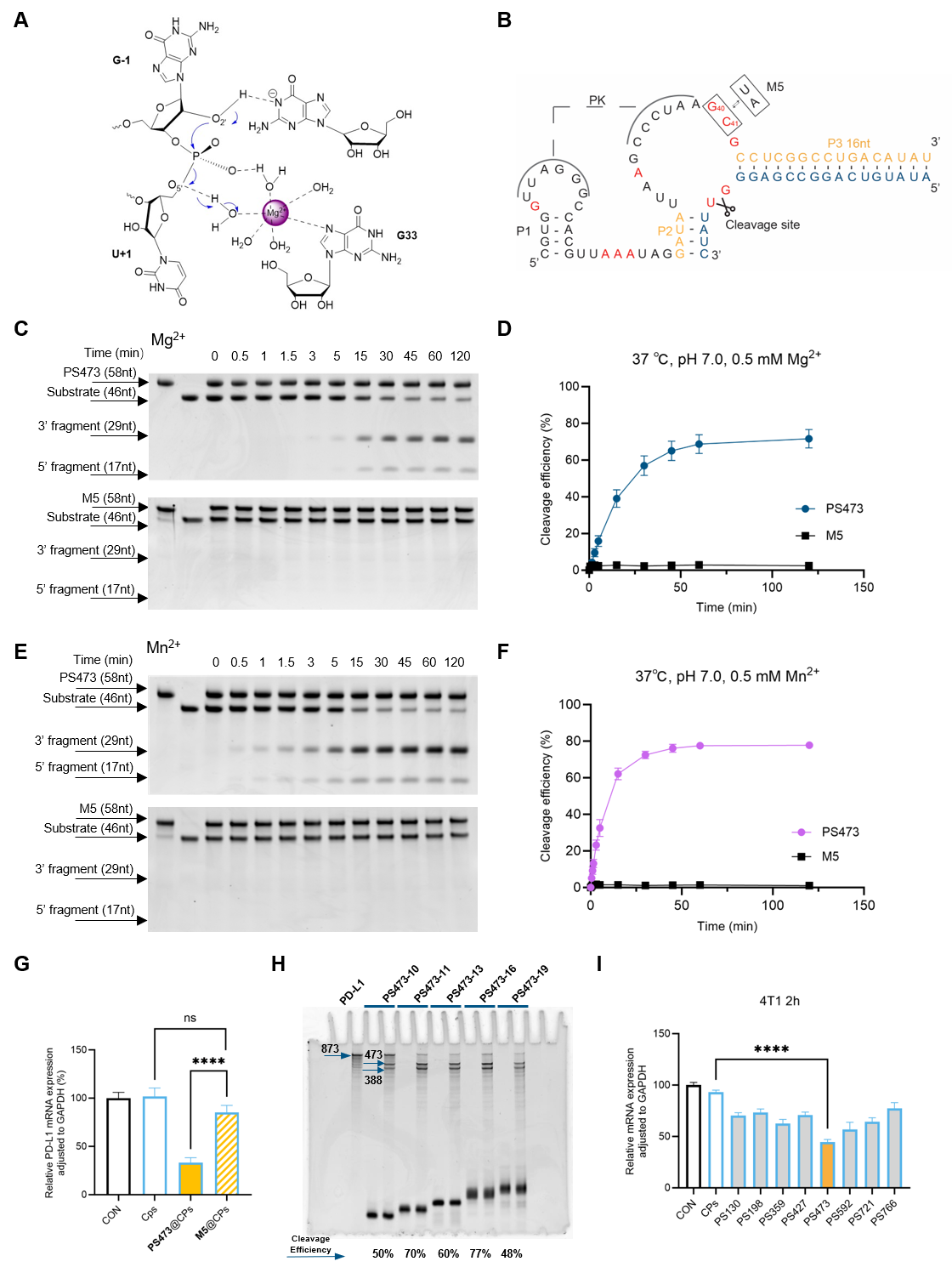
**

**Figure S1. Design, catalytic activity, and PD-L1 targeting validation of the PS473 ribozyme and its mutant variant M5.** (**A**) Proposed general acid–base catalytic mechanism of the Pistol ribozyme. A plausible general acid–base catalytic model for Pistol ribozyme–mediated RNA cleavage is illustrated. The 2′-hydroxyl group of the upstream nucleotide is activated by a guanine base acting as a general base, facilitating nucleophilic attack on the adjacent phosphorus atom and formation of a pentavalent transition state. Cleavage of the scissile phosphate is accompanied by protonation of the leaving 5′-oxygen, mediated by a Mg²⁺-coordinated water molecule acting as a general acid. The Mg²⁺ ion is shown coordinated by water molecules and nearby nucleobase functionalities (including guanine N7), stabilizing negative charge development during the reaction. Curved arrows indicate the direction of electron pair movement. (**B**) Secondary-structure schematic of the catalytically impaired **M5** mutant, showing the critical active-site mutations and the intact substrate-binding stem–loop region. (**C**) Time-course cleavage assay comparing **PS473** and **M5** activity using a 48-nt RNA substrate under physiological conditions (37 °C, pH 7.0, 0.5 mM Mg²⁺). Note: The cleavage gel image of PS473 under Mg²⁺ conditions corresponds to that shown in Figure 4E of the main text and is reused here for direct visual comparison. (**D**) Kinetic cleavage curves showing the time-dependent catalytic efficiency of **PS473** versus **M5**. **PS473** rapidly cleaves target RNA, whereas **M5** displays negligible activity. (**E**) Time-course cleavage assay comparing **PS473** and **M5** activity under identical conditions with 0.5 mM Mn²⁺. Note: The cleavage gel image of **PS473** under Mn²⁺ conditions corresponds to that shown in Figure 4E and is reused for the same comparative purpose. (**F**) Kinetic cleavage curves showing the time-dependent catalytic efficiency of **PS473** versus **M5**. **PS473** rapidly cleaves target RNA, whereas **M5** displays negligible activity. (**G**) Quantitative comparison of PD-L1 mRNA levels in B16F10 cells treated with PBS (CON), cationic polymer (CPs), **PS473**@CPs, and **M5**@CPs. **PS473** significantly reduces PD-L1 expression, whereas **M5** has no silencing effect. (**H**) In vitro cleavage assay of PD-L1 mRNA coding sequence (CDS) by various **PS473** ribozyme variants. PAGE analysis shows cleavage fragment bands, and quantification (below) indicates that **PS473** achieves the highest catalytic efficiency. (**I**) Relative suppression efficiency of Pistol ribozyme variants on PD-L1 mRNA expression in 4T1 cells. All data are presented as mean (± SD), n = 3. Statistical significance was assessed by two-tailed Student’s t-test: *****p* < 0.0001, ns = not significant.

**
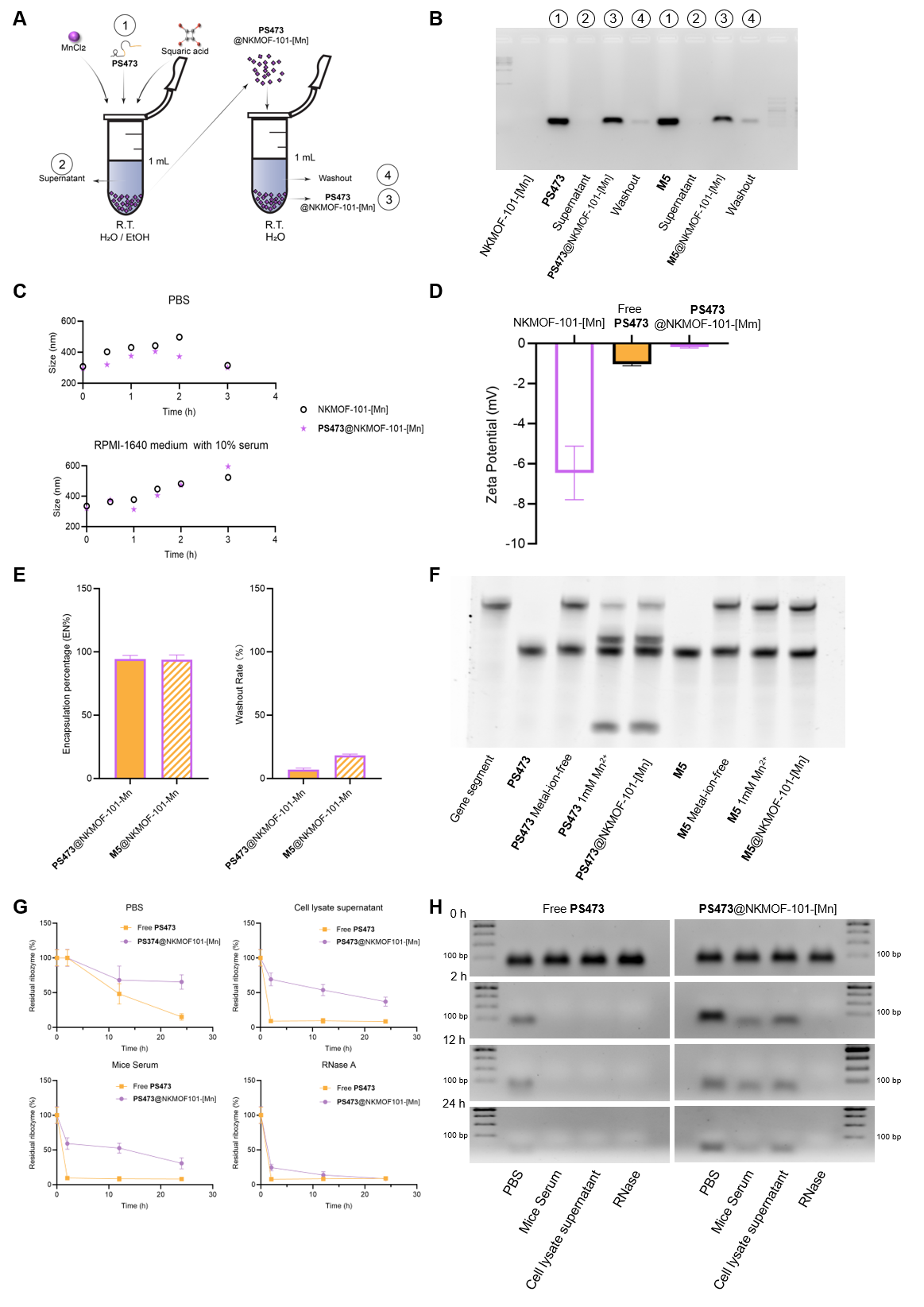
**

**Figure S2. Characterization and stability evaluation of PS473@NKMOF-101-[Mn] nanocomposites. (A)** Schematic illustration of the one-pot synthesis of **PS473**@NKMOF-101-[Mn] and **M5**@NKMOF-101-[Mn]. Ribozyme (**PS473** or **M5**), MnCl₂, and squaric acid were co-assembled in a H₂O/EtOH mixture at room temperature to form ribozyme-loaded NKMOF-101-[Mn] nanoparticles. After centrifugation and sequential washing, the pellet (material) and supernatant were collected for analysis. **(B)** Agarose gel electrophoresis of **PS473** and **M5** in different fractions (supernatant, material, and washout) confirms successful ribozyme loading onto NKMOF-101-[Mn] and removal of unbound RNA. **(C)** Dynamic light scattering (DLS) analysis showing the hydrodynamic size distribution of NKMOF-101-[Mn] and **PS473**@NKMOF-101-[Mn], revealing comparable particle diameters (~250–300 nm). **(D)** Zeta potential measurements of NKMOF-101-[Mn], Free **PS473**, and **PS473**@NKMOF-101-[Mn], indicating surface charge neutralization after ribozyme loading. **(E)** Quantification of encapsulation efficiency and unbound ribozyme content for **PS473**@NKMOF-101-[Mn] and **M5**@NKMOF-101-[Mn], confirming high loading efficiency and minimal residual RNA. **(F)** In vitro cleavage assay of PD-L1 mRNA fragment by various condition. **(G)** Quantitative analysis of **PS473** integrity in various biological matrices over time. **PS473**@NKMOF-101-[Mn] retained significantly higher stability than Free **PS473**, particularly in nuclease-rich environments such as serum and Cell lysate supematant solution. **(H)** Gel-based stability assay comparing degradation patterns of Free **PS473** and **PS473**@NKMOF-101-[Mn] in different matrices: PBS, mouse serum, cell lysate supernatant, and RNase A, indicating enhanced protection by the NKMOF-101-[Mn] carrier. All data are presented as mean (± SD), n = 3.


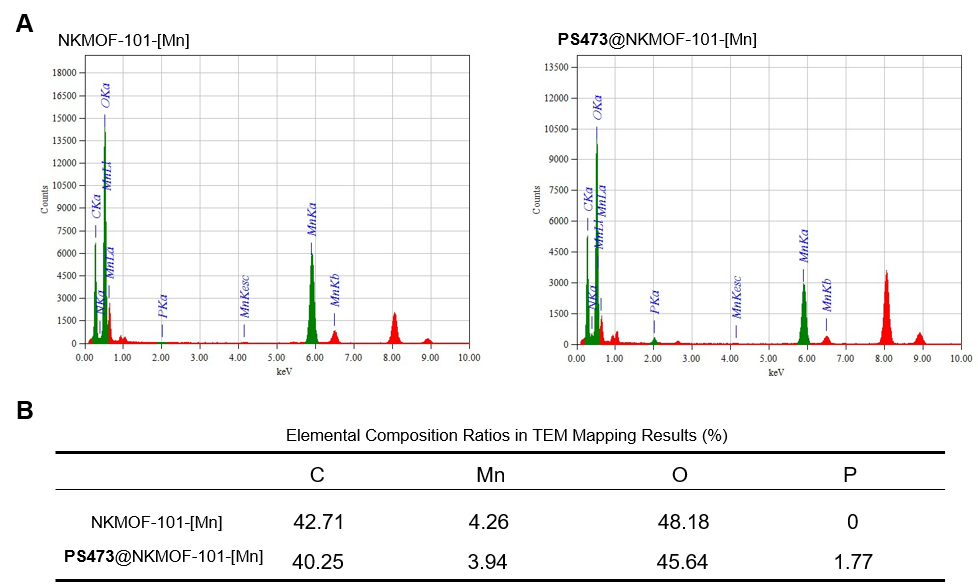


**Figure S3** (**A**) Energy - dispersive X - ray spectroscopy (EDS) analysis of NKMOF-101-[Mn] and **PS473**@NKMOF-101–[Mn]. (**B**) Elemental composition ratios of NKMOF-101-[Mn] and **PS473**@NKMOF-101-[Mn] in TEM mapping results. The table presents the composition ratios (%) of carbon (C), manganese (Mn), oxygen (O), and phosphorus (P) elements in NKMOF-101-[Mn] and **PS473**@NKMOF-101-[Mn] materials. The data show differences in elemental contents between the two materials, such as the presence of phosphorus in **PS473**@NKMOF-101-[Mn] while it is absent in NKMOF-101-[Mn].


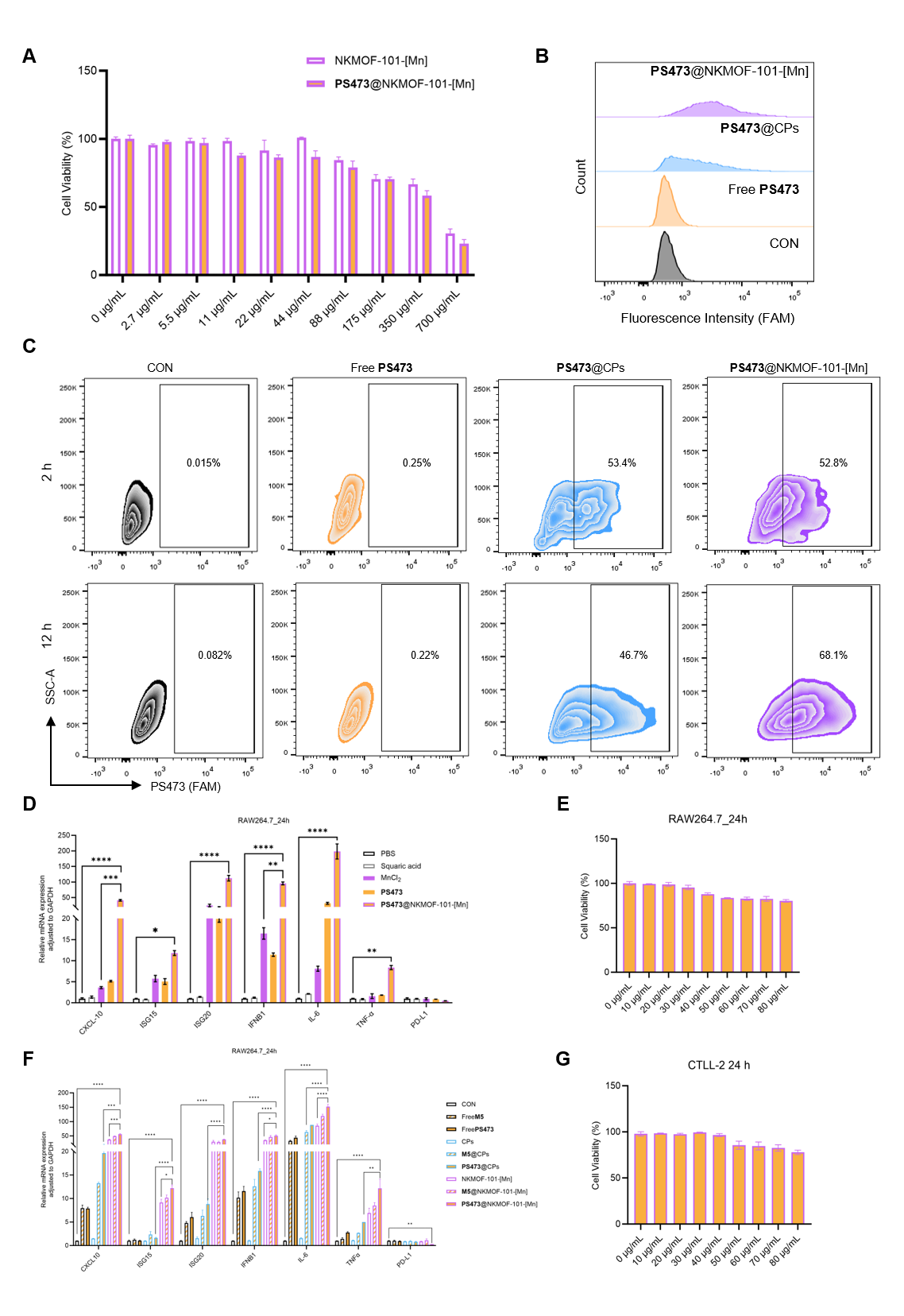


**Figure S4. Cellular uptake and cytocompatibility of PS473@NKMOF-101-[Mn]. (A)** Cell viability analysis of B16F10 cells treated with increasing concentrations of NKMOF-101-[Mn] or **PS473**@NKMOF-101-[Mn] for 24 h, as determined by CCK-8 assay. Both formulations exhibited dose-dependent cytotoxicity, with minimal impact observed at concentrations below 44 μg mL^-1^. **(B)** Quantitative flow cytometry analysis of FAM-labeled **PS473** uptake in B16F10 cells following 2 h incubation with Free **PS473**, **PS473**@CPs, or **PS473**@NKMOF-101-[Mn]. Compared to the Free **PS473** and CPs groups, NKMOF-101-[Mn]-mediated delivery resulted in robust intracellular fluorescence, indicating efficient cellular internalization. **(C)** Representative flow cytometric contour plots showing time-dependent intracellular accumulation of FAM-labeled **PS473** in B16F10 cells at 2 h and 12 h post-treatment. The **PS473**@NKMOF-101-[Mn] group showed high uptake (52.8%) at 2 h that persisted at 12 h (54.3%), in contrast to rapid decline in the **PS473**@CPs group (from 53.4% to 34.3%). Free **PS473** displayed negligible cellular uptake (<0.1%) at both time points. (**D**) qPCR analysis of innate-immune and interferon-stimulated genes (CXCL10, ISG15, ISG20, IFNB1, IL6, TNF) and PD-L1 in RAW264.7 macrophages treated with PBS, succinic acid (MOF ligand control), MnCl₂, **PS473**, or **PS473**@NKMOF-101-[Mn] for 24 h. **PS473**@NKMOF-101-[Mn] induces strong immunostimulation while reducing PD-L1. (**E**) Cell viability analysis of RAW264.7 cells treated with increasing concentrations of **PS473**@NKMOF-101-[Mn] for 24 h, as determined by CCK-8 assay. (**F**) qRT-PCR analysis of immune-related genes and PD-L1 in RAW264.7 macrophages after 24 h treatment with the indicated formulations. **PS473**@NKMOF-101-[Mn] induced the strongest immune activation and PD-L1 suppression. (**G**) Cell viability analysis of CTLL-2 cells treated with increasing concentrations of **PS473**@NKMOF-101-[Mn] for 24 h, as determined by CCK-8 assay. Both formulations exhibited dose-dependent cytotoxicity, with minimal impact observed at concentrations below 40 μg mL^-1^. All the data are presented as the mean (± SD), n = 3, with statistical significance indicated as ***p* < 0.01, ****p* < 0.001, and *****p* < 0.0001; ns = not significant.

**
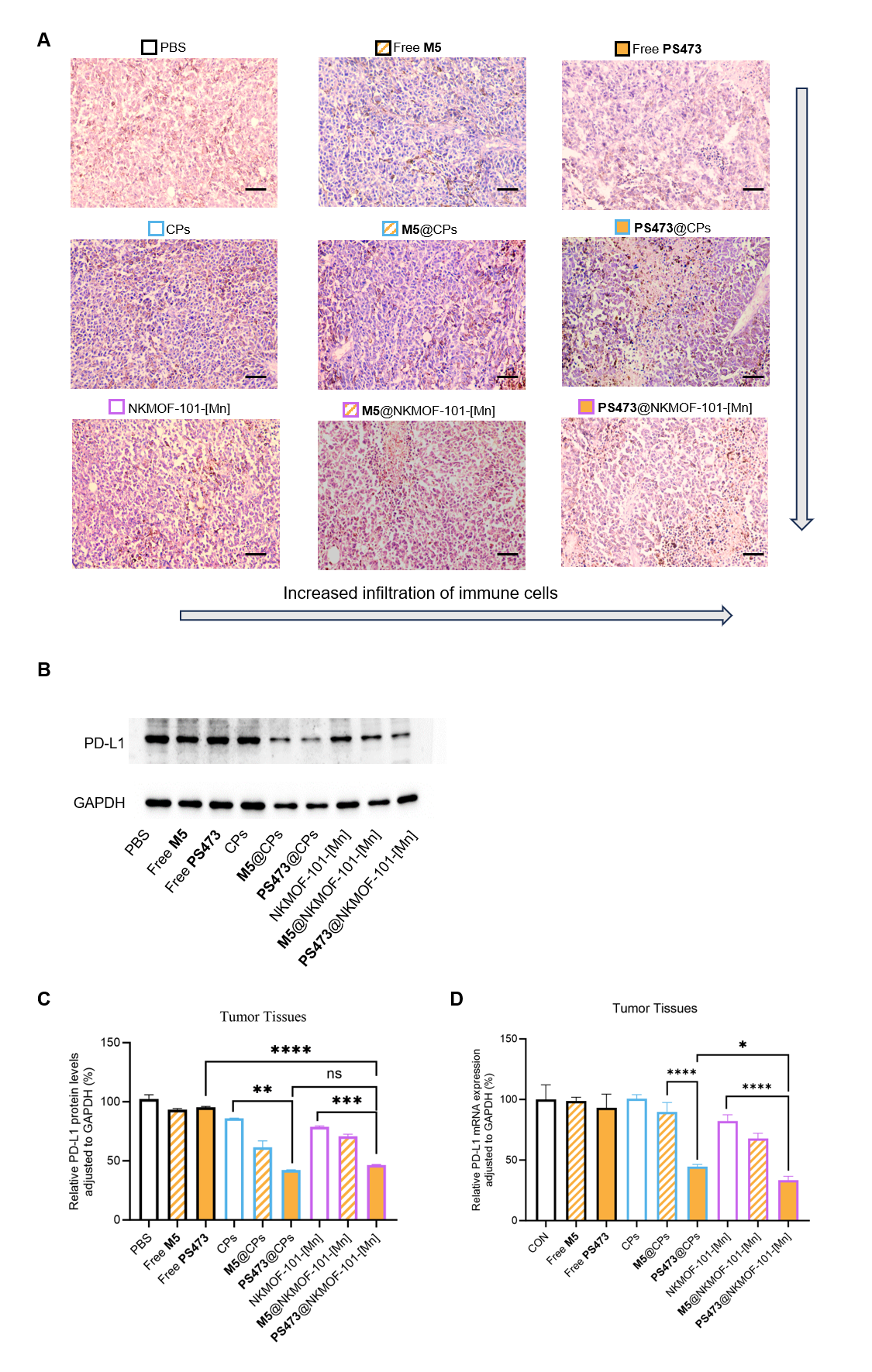
**

**Figure S5. (A)** Representative hematoxylin and eosin (H&E) staining of tumor sections from different treatment groups. Tumors treated with **PS473**@NKMOF-101-[Mn] displayed increased immune cell infiltration, cytoplasmic shrinkage, and nuclear condensation (pyknosis), suggestive of apoptosis and enhanced immune activation. The gradient arrow indicates increasing immune infiltration. Note: Staining intensity and color variation may reflect both biological responses and technical differences in sectioning or staining. **(B)** Representative Western blot analysis of PD-L1 protein levels in excised tumor tissues from each treatment group, with GAPDH used as a loading control. **(C, D)** Quantification of PD-L1 protein (C) and mRNA (D) expression levels normalized to GAPDH, revealing significant downregulation of PD-L1 in the **PS473**@NKMOF-101-[Mn] group compared to the Free **PS473**, **M5**, or carrier control groups in tumor tissues. Data are presented as mean (± SD), n = 3, with statistical significance indicated as ***p* < 0.01, ****p* < 0.001, *****p* < 0.0001, ns = not significant.


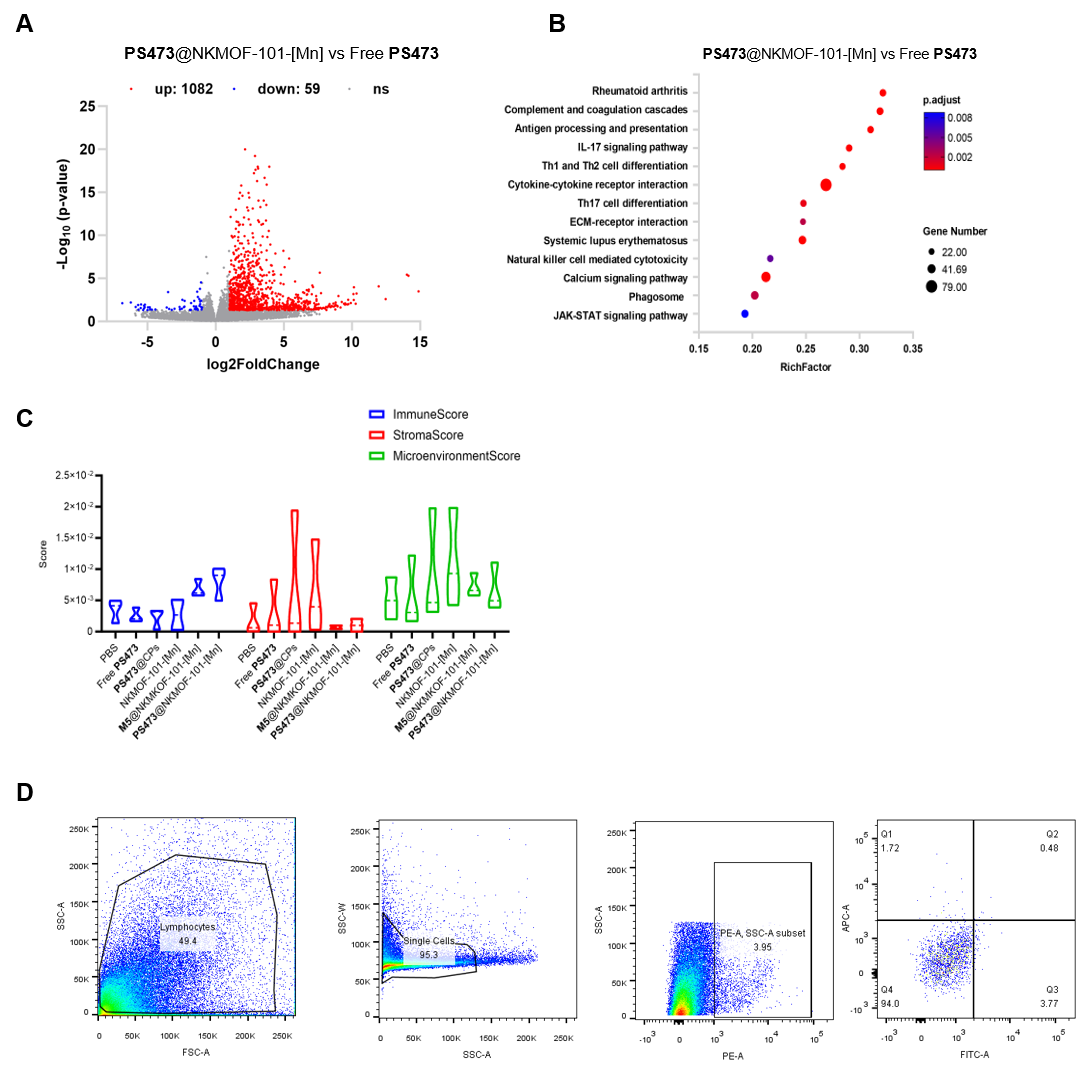


**Figure S6. Transcriptomic and tumor immune microenvironment analysis following PS473@NKMOF-101-[Mn] treatment. (A)** Volcano plot depicting differentially expressed genes (DEGs) between tumors treated with **PS473**@NKMOF-101-[Mn] and Free **PS473**. Red and blue dots represent significantly upregulated and downregulated genes, respectively (adjusted p < 0.05, |log₂ FoldChange| > 1); gray dots indicate non-significant genes. **(B)** KEGG pathway enrichment analysis of DEGs from panel (A). Immune- and inflammation-related pathways were significantly enriched in the **PS473**@NKMOF-101-[Mn] group. Circle size corresponds to the number of genes in each pathway, while color denotes the adjusted p-value of enrichment. **(C)** Quantitative comparison of ImmuneScore, StromaScore, and MicroenvironmentScore across different treatment groups, based on transcriptome deconvolution. ImmuneScore reflects the level of immune cell infiltration (e.g., T cells, B cells, dendritic cells); StromaScore indicates the relative abundance of stromal components (e.g., fibroblasts, extracellular matrix); and MicroenvironmentScore represents the combined contribution of both immune and stromal compartments within the tumor niche. **(D)** Representative flow cytometry gating strategy for tumor-infiltrating immune cell analysis.


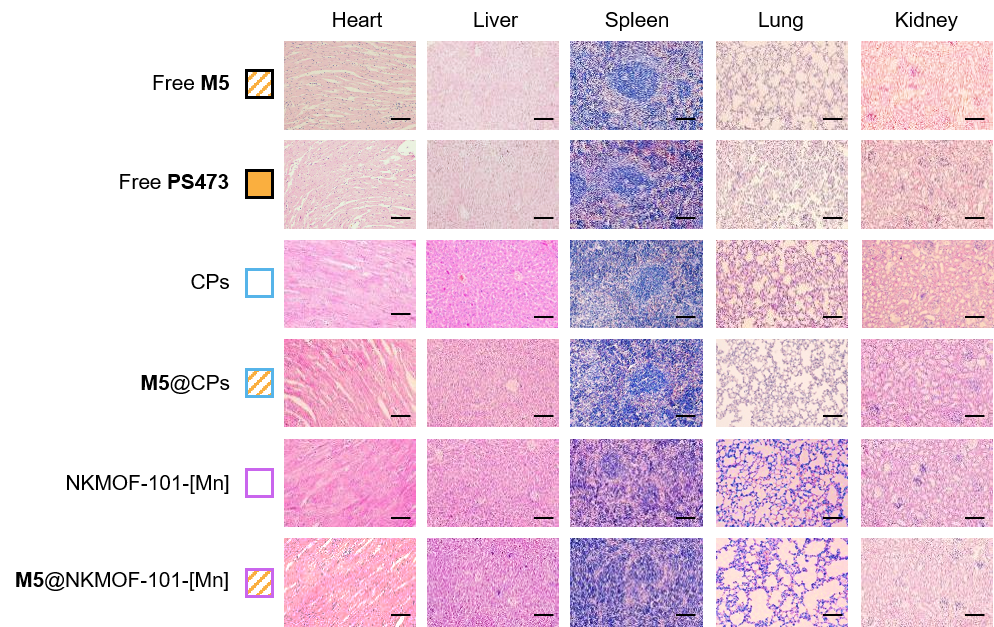


**Figure S7. Histopathological evaluation of major organs following treatment with various formulations.** Representative hematoxylin and eosin (H&E)-stained sections of the heart, liver, spleen, lung, and kidney collected from B16F10 tumor-bearing mice after treatment with Free **M5**, Free **PS473**, CPs, **M5**@CPs, NKMOF-101-[Mn], or **M5**@NKMOF-101-[Mn]. These panels complement Figure 9E by presenting the remaining treatment groups not shown in the main text. No significant signs of inflammation, necrosis, or tissue damage were observed in major organs across all groups, indicating favorable systemic biocompatibility. Scale bar = 100 μm.

Note: For presentation consistency, uniform global adjustments to brightness and color balance were applied equally to the entire images. No selective, local, or feature-specific modifications were performed, and no image content was added, removed, or altered. Each panel shown in Figures 9E and Supplementary Figure S7 corresponds to a unique animal sample; no histological image is duplicated across figures.

**Table S1:** **Nucleotide Sequences of PD-L1 mRNA-Targeting Pistol Ribozymes and Their Corresponding RNA Substrates**

Note: Detailed design methods can be found in the (Supplementary) Methods (section “Design of Pistol Ribozyme Variants”).

| RNA | Sequence |
| --- | --- |
| Pistol WT | CGUGGUUAGGGCCACGUUAAAUAGUUGCUUAAGCCCUAAGCGUUGAU ^[7]^ |
| PS38 | CGUGGUUAGGGCCACGUUAAAUAGAGUGUUAAGCCCUAAGCGAGCAGGCUGU |
| PS81 | CGUGGUUAGGGCCACGUUAAAUAGACGUUUAAGCCCUAAGCGAAGUCCUUUG |
| PS130 | CGUGGUUAGGGCCACGUUAAAUAGUUCUUUAAGCCCUAAGCGAGGGAAUCUG |
| PS198 | CGUGGUUAGGGCCACGUUAAAUAGACAAUUAAGCCCUAAGCGUGAAUCACUU |
| PS359 | CGUGGUUAGGGCCACGUUAAAUAGCCGCUUAAGCCCUAAGCGCACCGUAGCU |
| PS388 | CGUGGUUAGGGCCACGUUAAAUAGAUUGUUAAGCCCUAAGCGUUUCAGCGUG |
| PS427 | CGUGGUUAGGGCCACGUUAAAUAGAUCCUUAAGCCCUAAGCGGGAAAUUCUC |
| PS473 | CGUGGUUAGGGCCACGUUAAAUAGGAUAUUAAGCCCUAAGCGCCUCGGCCUG |
| PS538 | CGUGGUUAGGGCCACGUUAAAUAGGGUGUUAAGCCCUAAGCGACUUCUCUUC |
| PS592 | CGUGGUUAGGGCCACGUUAAAUAGGUUGUUAAGCCCUAAGCGCCUCAGACUG |
| PS623 | CGUGGUUAGGGCCACGUUAAAUAGACGUUUAAGCCCUAAGCGAGUAGAAAAC |
| PS721 | CGUGGUUAGGGCCACGUUAAAUAGAAGCUUAAGCCCUAAGCGCCAGUGAGUC |
| PS766 | CGUGGUUAGGGCCACGUUAAAUAGGAGGUUAAGCCCUAAGCGCGUGGACACU |
| PS815 | CGUGGUUAGGGCCACGUUAAAUAGCGCCUUAAGCCCUAAGCGAUUUCUCCAC |
| PD-L1 mRNA Substrate  (873 nt) | AUGAGGAUAUUUGCUGGCAUUAUAUUCACAGCCUGCUGUCACUUGCUACGGGCGUUUACUAUCACGGCUCCAAAGGACUUGUACGUGGUGGAGUAUGGCAGCAACGUCACGAUGGAGUGCAGAUUCCCUGUAGAACGGGAGCUGGACCUGCUUGCGUUAGUGGUGUACUGGGAAAAGGAAGAUGAGCAAGUGAUUCAGUUUGUGGCAGGAGAGGAGGACCUUAAGCCUCAGCACAGCAACUUCAGGGGGAGAGCCUCGCUGCCAAAGGACCAGCUUUUGAAGGGAAAUGCUGCCCUUCAGAUCACAGACGUCAAGCUGCAGGACGCAGGCGUUUACUGCUGCAUAAUCAGCUACGGUGGUGCGGACUACAAGCGAAUCACGCUGAAAGUCAAUGCCCCAUACCGCAAAAUCAACCAGAGAAUUUCCGUGGAUCCAGCCACUUCUGAGCAUGAACUAAUAUGUCAGGCCGAGGGUUAUCCAGAAGCUGAGGUAAUCUGGACAAACAGUGACCACCAACCCGUGAGUGGGAAGAGAAGUGUCACCACUUCCCGGACAGAGGGGAUGCUUCUCAAUGUGACCAGCAGUCUGAGGGUCAACGCCACAGCGAAUGAUGUUUUCUACUGUACGUUUUGGAGAUCACAGCCAGGGCAAAACCACACAGCGGAGCUGAUCAUCCCAGAACUGCCUGCAACACAUCCUCCACAGAACAGGACUCACUGGGUGCUUCUGGGAUCCAUCCUGUUGUUCCUCAUUGUAGUGUCCACGGUCCUCCUCUUCUUGAGAAAACAAGUGAGAAUGCUAGAUGUGGAGAAAUGUGGCGUUGAAGAUACAAGCUCAAAAAACCGAAAUGAUACACAAUUCGAGGAGACGUAA |
| Substrate (46nt) | AUAUGUCAGGCCGAGGGUUAUCUAGGAUCUGUCUAGUCCAGAUCAC |

**Table S2: Inactivated Mutant Sequences of PS473 Ribozyme**

| Ribozyme | Ribozyme sequence |
| --- | --- |
| MUTATION_5 (**M5**) | CGUGGUUAGGGCCACGUUAAAUAGGAUAUUAAGCCCUAAUAGCCUCGGCCUGACAUAU |

**Table S3: Sequences of Pistol Ribozymes with Different P3 Lengths Targeting Specific Sites on PD-L1 mRNA**

| Ribozyme | Ribozyme Sequence |
| --- | --- |
| PS473_7 | CGUGGUUAGGGCCACGUUAAAUAGGAUAUUAAGCCCUAAGCGCCUCGGCGCCCUAAGCGCCUCGGC |
| PS473_13 | CGUGGUUAGGGCCACGUUAAAUAGGAUAUUAAGCCCUAAGCGCCUCGGCGCCCUAAGCGCCUCGGCCUGACA |
| PS473_14 | CGUGGUUAGGGCCACGUUAAAUAGGAUAUUAAGCCCUAAGCGCCUCGGCGCCCUAAGCGCCUCGGCCUGACAUA |
| PS473_15 | CGUGGUUAGGGCCACGUUAAAUAGGAUAUUAAGCCCUAAGCGCCUCGGCGCCCUAAGCGCCUCGGCCUGACAUAU |
| PS473_16 | CGUGGUUAGGGCCACGUUAAAUAGGAUAUUAAGCCCUAAGCGCCUCGGCGCCCUAAGCGCCUCGGCCUGACAUAUA |
| PS473_17 | CGUGGUUAGGGCCACGUUAAAUAGGAUAUUAAGCCCUAAGCGCCUCGGCGCCCUAAGCGCCUCGGCCUGACAUAUAA |
| PS473_18 | CGUGGUUAGGGCCACGUUAAAUAGGAUAUUAAGCCCUAAGCGCCUCGGCGCCCUAAGCGCCUCGGCCUGACAUAUAUA |
| PS473_19 | CGUGGUUAGGGCCACGUUAAAUAGGAUAUUAAGCCCUAAGCGCCUCGGCGCCCUAAGCGCCUCGGCCUGACAUAUAUAG |

**Table S4 Primer sequences used in RT-qPCR analysis**

| Primers | Sequence |
| --- | --- |
| CXCL-10 | F: TCACTCCCCTTTACCCAG |
|  | R: GTCGCACCTCCACATAGCTT |
| ISG15 | F: AAGCAGCCAGAAGCAGACTC |
|  | R: TAAGACCGTCCTGGAGCACT |
| ISG20 | F: GCCGAGAAGTGGAAACAGAG |
|  | R: CTCGGGTCGGATGTACTTGT |
| IFNB1 | F: CCCTATGGAGATGACGGAGA |
|  | R: CTGTCTGCTGGTGGAGTTCA |
| IL-6 | F: AGTTGCCTTCTTGGGACTGA |
|  | R: TCCACGATTTCCCAGAGAAC |

**References**

1. Lilley, D.M.J. (2019). Classification of the nucleolytic ribozymes based upon catalytic mechanism. F1000Res 8. https://doi.org/10.12688/f1000research.19324.1.

2. Ekesan, Ş., and York, D.M. (2022). Who stole the proton? Suspect general base guanine found with a smoking gun in the pistol ribozyme. Org Biomol Chem 20, 6219-6230. https://doi.org/10.1039/d2ob00234e.

3. Gaines, C.S., Piccirilli, J.A., and York, D.M. (2020). The L-platform/L-scaffold framework: a blueprint for RNA-cleaving nucleic acid enzyme design. Rna 26, 111-125. https://doi.org/10.1261/rna.071894.119.

4. Lihanova, Y., and Weinberg, C.E. (2021). Biochemical analysis of cleavage and ligation activities of the pistol ribozyme from Paenibacillus polymyxa. RNA Biol 18, 1858-1866. https://doi.org/10.1080/15476286.2021.1874706.

5. Yoon, S., Ollie, E., York, D.M., et al. (2023). Rapid Kinetics of Pistol Ribozyme: Insights into Limits to RNA Catalysis. Biochemistry 62, 2079-2092. https://doi.org/10.1021/acs.biochem.3c00160.

6. Egger, M., Bereiter, R., Mair, S., et al. (2022). Scaling Catalytic Contributions of Small Self-Cleaving Ribozymes. Angew Chem Int Ed Engl 61, e202207590. https://doi.org/10.1002/anie.202207590.

7. Ren, A., Vušurović, N., Gebetsberger, J., et al. (2016). Pistol ribozyme adopts a pseudoknot fold facilitating site-specific in-line cleavage. Nat Chem Biol 12, 702-708. https://doi.org/10.1038/nchembio.2125.

8. Harris, K.A., Lünse, C.E., Li, S., et al. (2015). Biochemical analysis of pistol self-cleaving ribozymes. Rna 21, 1852-1858. https://doi.org/10.1261/rna.052514.115.
